# Late Development of FcεRγ^neg^ Adaptive Natural Killer Cells Upon Human Cytomegalovirus Reactivation in Umbilical Cord Blood Transplantation Recipients

**DOI:** 10.3389/fimmu.2018.01050

**Published:** 2018-05-15

**Authors:** Letizia Muccio, Michela Falco, Alice Bertaina, Franco Locatelli, Francesco Frassoni, Simona Sivori, Lorenzo Moretta, Alessandro Moretta, Mariella Della Chiesa

**Affiliations:** ^1^Dipartimento di Medicina Sperimentale, Università degli Studi di Genova, Genova, Italy; ^2^IRCCS Istituto Giannina Gaslini, Dipartimento dei Laboratori di Ricerca, Genova, Italy; ^3^IRCCS Ospedale Pediatrico Bambino Gesù, Dipartimento di Oncoematologia e Terapia Cellulare e Genica, Rome, Italy; ^4^Dipartimento di Scienze Pediatriche, Università degli Studi di Pavia, Pavia, Italy; ^5^Centro di Eccellenza per le Ricerche Biomediche, Università degli Studi di Genova, Genova, Italy; ^6^IRCCS Ospedale Pediatrico Bambin Gesù, Area di Ricerca Immunologica, Rome, Italy

**Keywords:** adaptive natural killer cells, FcεRγ, human cytomegalovirus reactivation, NKG2C-CD57, UCB transplant, ADCC

## Abstract

In human natural killer (NK) cells, human cytomegalovirus (HCMV) has been shown to be a driving force capable of inducing the expansion of a highly differentiated NKG2C^+^CD57^+^ subset, persisting over time in both HCMV^+^ healthy subjects and umbilical cord blood transplantation (UCBT) recipients experiencing HCMV viral reactivation. In HCMV^+^ healthy subjects, such expanded NK-cells are characterized by epigenetic modifications that modulate their phenotypic and functional characteristics. In particular, an enhanced ADCC activity is detectable in NK cells lacking the signaling protein FcεRγ. Timing and mechanisms involved in the acquisition of HCMV-induced, adaptive-like features by NK cells are currently unknown. In this study, we investigated the *de novo* acquisition of several adaptive features in NK cells developing after UCBT by monitoring NK-cell differentiation for at least 2 years after transplant. In UCBT recipients experiencing HCMV reactivation, a rapid phenotypic reconfiguration occurred resulting in the expected expansion of CD56^dim^ NKG2C^+^CD57^+^ NK cells. However, while certain HCMV-driven adaptive hallmarks, including high KIR, LILRB1, CD2 and low/negative NKG2A, Siglec-7, and CD161 expression, were acquired early after UCBT (namely by month 6), downregulation of the signaling protein FcεRγ was detected at a later time interval (i.e., by month 12). This feature characterized only a minor fraction of the HCMV-imprinted NKG2C^+^CD57^+^ CD56^dim^ NK cell subset, while it was detectable in higher proportions of CD57^+^ NK cells lacking NKG2C. Interestingly, in patients developing a hyporesponsive CD56^−^CD16^bright^ NK-cell subset, FcεRγ downregulation occurred in these cells earlier than in CD56^dim^ NK cells. Our data suggest that the acquisition of a fully “adaptive” profile requires signals that may lack in UCBT recipients and/or longer time is needed to obtain a stable epigenetic reprogramming. On the other hand, we found that both HCMV-induced FcεRγ^neg^ and FcεRγ^+^ NK cells from these patients, display similar CD107a degranulation and IFN-γ production capabilities in response to different stimuli, thus indicating that the acquisition of specialized effector functions can be achieved before the “adaptation” to HCMV is completed. Our study provides new insights in the process leading to the generation of different adaptive NK-cell subsets and may contribute to develop new approaches for their employment as novel immunotherapeutic tools.

## Introduction

Natural killer (NK) cells are a lymphoid population belonging to the innate immunity capable of providing rapid responses against tumors and viral infections without prior sensitization (1). Their activity is regulated by germ-line encoded inhibitory and activating receptors (2–4) and can be modulated by cytokines/chemokines, PAMPS, and education mechanisms (5).

It is known since several years that viral infections, primarily those due to human cytomegalovirus (HCMV), shape the NK cell receptor repertoire and influence NK cell development and function (6, 7). In particular, initial studies revealed that HCMV infection may induce a stable expansion of a NK cell subset expressing the activating receptor CD94/NKG2C and displaying a differentiated phenotype mainly NKG2A^−^LILRB1^+^KIR^+^CD57^+^ in healthy individuals (8). It was suggested that the NKG2C receptor could play a crucial role in favoring this expansion by interacting with ligands on HCMV-infected target cells (9). In particular, the expression of non-classic HLA-class I molecule HLA-E, which represents the only known ligand for NKG2C (10), is necessary to induce proliferation of NKG2C^+^ NK cell (11). Subsequently, based on results emerging in murine models of CMV infection (12), the imprinting exerted by HCMV on NK cells has been further investigated and reinterpreted, revealing unexpected adaptive features displayed by HCMV-induced NK cells, namely (oligo)clonal expansion, enhanced effector function, longevity, as well as epigenetic modifications (13, 14). Thus, the so called memory-like NKG2C^+^CD57^+^NK cell subset was shown to display an epigenetic remodeling at the IFN-γ locus, similar to that found in effector memory T cells, which is likely to be responsible for the enhanced IFN-γ production upon target cell recognition detectable in NKG2C^+^ NK cells (15). Moreover, in HCMV^+^ healthy individuals, a variable proportion of NK cells, mainly (but not exclusively) belonging to the NKG2C^+^ subset, were characterized by a decreased expression of signaling molecules, such as the adaptor proteins FcεRγ and EAT-2, and the tyrosine kinase Syk. In addition, these cells displayed a lower expression of the transcription factor PLZF, which is involved in the regulation of epigenetic modifications (e.g., DNA hypo-/hypermethylation) that, in turn, influence the expression of receptors and adaptor proteins (16, 17). These NK cells, showing hallmarks of adaptive immunity, are now often referred to as adaptive NK cells (18–20). Among the above adaptive features, the lack of FcεRγ was more frequently detected (16, 17). It has been proposed that this event may favor more efficient ADCC responses against Ab-coated HCMV-infected targets by such adaptive FcεRγ^neg^ NK cells (17, 21, 22). However, the precise mechanisms regulating the generation of adaptive NK cells, as well as the time when different adaptive traits are acquired after HCMV infection, are not fully elucidated.

The imprinting induced by HCMV infection may vary among individuals, but it is particularly strong in immunocompromised subjects, such as patients undergoing hematopoietic stem cell transplantation (HSCT). Indeed, following HCMV infection/reactivation, a rapid reconstitution of a differentiated KIR^+^NKG2A^−^NKG2C^+^CD57^+^ NK cell subset, which expands and persists over time, was initially detected in patients receiving umbilical cord blood transplantation (UCBT) (23, 24). Thus, NK cells generated after HSCT appeared to undergo a viral-induced (oligo) clonal expansion and to be long living. These findings that contributed to the concept of adaptive NK cells, prompted us to investigate the emergence of the HCMV-induced adaptive features in NK cells reconstituting after UCBT. This transplantation setting, in which the graft is composed of HSC precursors and *naive* lymphoid cells, allows the identification of *de novo* generated adaptive NK cells. By focusing on some of the most relevant adaptive characteristics (FcεRγ, PLZF, and selected surface receptors expression), we could monitor their acquisition by NK cells undergoing differentiation in patients experiencing HCMV reactivation in an ample time window after UCBT (1–24 m).

We show that, despite a remarkable expansion of mature NKG2C^+^CD57^+^ NK cells showing several HCMV-driven hallmarks (high KIR, LILRB1, CD2, low/negative NKG2A, Siglec-7, CD161), the downregulation of the signaling protein FcεRγ (a crucial adaptive trait) appeared late after transplantation. In addition, FcεRγ downregulation occurred only in a minor fraction of the HCMV-imprinted NKG2C^+^CD57^+^ CD56^dim^ NK cell subset, while it was detectable in slightly higher proportions of mature NKG2C^−^CD57^+^ NK cells. This finding suggests that the acquisition of a fully adaptive signature requires either signals that may lack in UCBT recipients or longer times to obtain a stable epigenetic reprogramming.

## Materials and Methods

### Patients, Samples, and Ethical Statements

Seventeen patients with hematological malignancies (7 children and 10 adults), mostly acute myeloid leukemia, were included in this study. All patients received UCBT at the Bambino Gesù Children’s Hospital, Rome, Italy (pediatric patients) or at the San Martino Hospital, Genoa, Italy (adult patients). Either patients or their parents gave their informed consent to participation in this study, which was approved by the Azienda Ospedaliera Universitaria San Martino (Genoa, Italy), by the University of Genoa and by the Bambino Gesù Children’s Hospital (Rome, Italy) ethics committees and was conducted in accordance with the tenets of the Declaration of Helsinki. Details on patients’ clinical characteristics are summarized in Table S1 in Supplementary Material. All patients received a combination of cyclosporine-A (Novartis Pharma), mycophenolate mofetil (Roche), and an antithymocyte globulin (Genzyme) as graft-versus-host disease (GvHD) prophylaxis. Cyclosporine-A was started intravenously from day −7 before transplantation at a daily dose of 1 mg/kg recipient body weight. The dose of cyclosporine-A was adjusted to maintain a serum trough level between 150 and 300 µg/L. After engraftment, cyclosporine-A was given orally and, starting from day +90 after UCBT, progressively tapered until discontinuation. Mycophenolate mofetil was administered at a dosage of 15 mg/kg twice a day from day 1 to day 28 after transplantation. Antithymocyte globulin was given before transplantation at a dose of 2–3 mg/kg on days −3 and −2. No patients received steroids for GvHD prophylaxis.

Peripheral blood samples were collected from patients at 1, 6, 12, and 24 months after transplantation. Peripheral blood mononuclear cells (PBMC) were separated from blood samples by Ficoll-Hypaque gradients (Sigma-Aldrich, St. Louis, MO, USA), frozen, and subsequently thawed for flow cytometry analyses and functional assays. Three HCMV-reactivating patients received UCBT from donors carrying *NKG2C* gene homozygous deletion (see Results); therefore, NK cells isolated from these patients were analyzed separately and are not included in those assays based on NKG2C expression evaluation.

Peripheral blood mononuclear cells collected from adult healthy donors (HD) and UCB units provided by Centro Trasfusionale Azienda Ospedaliera Universitaria San Martino (Genoa, Italy), were used as controls.

### HCMV Serology and Therapy

Human cytomegalovirus serology was assessed prior to transplantation using enzyme-linked immunoassay for virus-specific immunoglobulin IgM and IgG. Patients were monitored for HCMV reactivation in blood by determination of DNAemia twice a week from day 0 until discharge from the hospital, and then once a week for the first 3 months. Subsequently, patients were monitored for HCMV at time of control medical visits or in the presence of clinical symptoms suggestive of HCMV infection. HCMV reactivation was monitored in the posttransplant period by antigenemia determination, counting HCMV pp65^+^ cells/2 × 10^5^ PMN cells. Pre-emptive therapy was based on administration of i.v. ganciclovir (5 mg/kg twice a day), replaced by foscarnet (90 mg/kg twice a day) in case of ganciclovir-induced neutropenia (less than 0.5 × 10^9^ neutrophils/L) or sustained increase of HCMV levels in blood during therapy with ganciclovir. Antiviral treatment was discontinued after virus clearance from blood, defined as two consecutive negative results. Episodes of HCMV relapse were treated similarly. Details on HCMV reactivation episodes and serology are reported in Table S1 in Supplementary Material.

### Monoclonal Antibodies (mAbs) and Flow Cytometry

The following mAbs, produced in our laboratory, were used in this study: C127 (IgG1, anti-CD16), MA311 (IgG1, anti-CD161), MAR206 (IgG1, anti-CD2), PP35 (IgG1, anti-CD244), Z199 (IgG2b, anti-NKG2A), QA79 (IgG1, anti-Siglec-7), DF200 (IgG1, anti-KIR2DL1/S1/L2/S2/L3/S5), AZ158 (IgG2a, anti-KIR3DL1/S1/L2), 11PB6 (IgG1, anti-KIR2DL1 and KIR2DS1), GL183 (IgG1, anti-KIR2DL2/L3/S2), FES172 (IgG2a, anti-KIR2DS4), and Z27 (IgG1, anti-KIR3DL1/S1). F278 (IgG1, anti–LILRB1/ILT-2) was kindly provided by Dr. D. Pende. The following commercial antibodies were used: anti-NKG2C (134522 clone), anti-KIR2DL1/KIR2DS5-FITC or -APC (143211 clone) were purchased from R&D Systems (Abingdon, UK); anti-CD56-PC7 (N901 clone) and anti-NKG2A-APC (z199 clone) were purchased from Beckman Coulter, Immunotech (Marseille, France); anti–CD3-VioGreen (BW264/56 clone), anti-CD19-VioGreen (LT19 clone), anti-CD14-Viogreen (TUK4 clone), anti-CD33-Viogreen (REA775 clone), anti-CD57-Vioblue (TB03 clone), biotin-conjugated anti-NKG2A (REA110 clone), anti-KIR2DL2/L3-S2-APC (DX27 clone), anti-KIR2DL1/S1-FITC (11PB6 clone), anti-KIR3DL1-FITC (DX9 clone), and anti-biotinPerCPVio700 mAbs were purchased from Miltenyi Biotec (Bergisch Gladbach, Germany); anti-CD16-PerCP-Cy5.5 (clone 3G8), anti-KIR2DL2/L3-S2-FITC (CH-L clone), anti-CD161-PE (C3H clone), anti-CD107a-APC-H7 (anti-LAMP1), anti-IFN-γ-PE, and anti-Syk-PE were from BD Biosciences (San Diego, CA, USA); MilliMark anti-FcεRγsubunit-FITC was purchased from Sigma-Aldrich; anti-Syk-FITC, anti-CD3ζ-PE, anti-PLZF-PE, and anti-T-bet-PE were purchased from eBioscience Inc. (San Diego, CA, USA); anti-human HLA-E (3D12 clone, IgG1) was purchased from Abnova (Taipei, Taiwan). Goat anti-mouse isotype-specific secondary reagents were purchased from Southern Biotech (Birmingham, AL, USA) and Jackson ImmunoResearch Laboratories (Suffolk, UK). Appropriated isotype controls were used when fluorochrome-labeled mAbs were employed.

Natural killer cell phenotype and effector functions were mainly analyzed on thawed PBMC, gating NK cells by physical parameters and by the combined use of anti-CD56, anti-CD16, anti-CD3, anti-CD19, anti-CD14 mAbs. To evaluate the presence of the CD56^−^CD16^bright^ NK cell subset, anti-CD33 mAb has been included in the gating strategy. The whole CD56^dim^ NK cell subset or the different NKG2C/CD57 CD56^dim^ subsets were evaluated whenever indicated (see gating strategy in Figure S1 in Supplementary Material). For intracellular cytofluorimetric analyses, cells were fixed and permeabilized with Foxp3 permeabilization buffer Kit (Miltenyi Biotec) according to the manufacturer’s instructions.

Cytofluorimetric analyses were performed on eight-colors BD FACSVerse (Becton Dickinson, Mountain View, CA, USA), and data were analyzed by FACSuite software version 1.0.5.

### Functional Assays

The medium used throughout the experiments was RPMI 1640, supplemented with 2 mM l-glutamine, 1% penicillin–streptomycin–neomycin mixture, and 10% heat-inactivated FCS. PBMC were cultured overnight in the presence of rhIL-15 (Peprotech, London, UK) at a final concentration of 10 ng/mL, washed, and used in the different assays. In reverse Ab-dependent cellular functional assays, PBMC from UCBT patients and HCMV^+^ HD were incubated with the FcγR^+^ p815 murine mastocytoma cell line, either in the presence or in the absence of mAbs (5 or 0.1 µg/mL for anti-CD16 and 0.05 µg/mL for anti-NKG2C) specific for different surface receptors indicated in the text and figures at an E:T ratio of 1:1 (where effector cells represent the effective number of PB-NK cells) for 4 h in culture medium supplemented with anti-CD107a-APC-H7 mAb and Golgi Stop (BD Biosciences Pharmingen) that was added for the last 3 h of culture. Cells were stained with anti-NKG2C and anti-NKG2A-biotin mAbs for 35 min on ice. Thereafter, cells were washed and stained with appropriate secondary reagents and anti-CD56-PC7, anti-CD57 vioblue and anti-CD3 and anti-CD19-viogreen for 35 min on ice. After staining, cells were fixed and permeabilized with Foxp3 permeabilization buffer Kit. IFN-γ production was detected on FcεRγ^+^ and FcεRγ^neg^ NK-cell subsets by subsequent intracellular staining with anti-IFN-γ-PE and anti-FcεRγ-FITC and cytofluorimetric analysis. The percentage of positive cells was calculated by subtracting the baseline CD107a or IFN-γ expression in control cultures (i.e., without stimuli from targets).

In different assays, degranulation and IFN-γ production were analyzed on FcεRγ^+^ and FcεRγ^neg^ NK cell subets from PBMC stimulated with Raji cell line, previously coated or not with the human anti-CD20 Rituximab (1 µg/mL), at an E:T ratio of 1:1, for 4 h in culture medium supplemented with anti-CD107a-APC-H7 mAb and Golgi Stop in the last 3 h of culture. Cells were then stained, permeabilized, and analyzed as described above.

### CFSE Proliferation Assays

Peripheral blood mononuclear cells were labeled with the intracellular fluorescent dye 5(6)-carboxyfluorescein diacetate succinimidyl ester (CFSE, Molecular Probes, Life Technologies, OR, USA). Briefly, extensively washed PBMC were resuspended in RPMI (10^7^/mL) and incubated for 10 min at 37°C with CFSE (2.5 µM). After three washes, CFSE-labeled PBMC were cultured either in the presence or in the absence of irradiated lymphoblastoyd 721.221wt cell line (221wt) or 721.221AEH cell line [221AEH, kind gift from prof. Miguel Lopez-Botet and dr. Aura Muntasell, originally developed by Geraghty D. et al. (25)] that has been transfected with a hybrid HLA-E containing the HLA-A2 signal sequence, at an E:T ratio (NK:221) of 5:1. Cocultures were kept in culture medium containing rhIL-2 (Proleukin, Chiron Corp., Emeryville, CA, USA) at a final concentration of 50 UI/mL. FACS analyses were performed at 7, 10, and 14 days as described above. NK cells cultured in the presence of 221AEH were analyzed for FcεRγ expression at 14th day of culture (i.e., when NK cells, upon proliferation, had undergone the complete dilution of the CFSE dye). After surface staining, cells were fixed and permeabilized as described above and subsequently stained with anti-FcεRγ-FITC mAb.

### Genotyping of NKG2C-Deleted Haplotype

DNA of the tested samples was extracted using QIAamp DNA Blood Mini kit (Qiagen, Hilden, Germany). The presence or absence of the NKG2C gene was determined as previously described with minor modifications (26). In particular, in each PCR reaction three sets of primers are present: the two primer pairs that allow discriminating between the haplotypes in which the NKG2C gene was present and those in which it was absent, in combination with the set specific for the NKG2A gene. The PCR conditions consisted of an initial denaturation at 95°C for 2 min, followed by 35 cycles: 30 s at 95°C, 30 s at 55°C, and 30 s at 72°C.

### Statistical Analysis

Wilcoxon–Mann–Whitney nonparametric tests were employed. The statistical significance (*p* value) is indicated (*p < 0.05; **p < 0.01; ***p < 0.001). Median fluorescence intensity (MFI) values were normalized before calculating statistical significance. Graphic representations and statistical analyses were performed with GraphPad Prism 6 (GraphPad Software, La Jolla, CA, USA).

## Results

### FcεRγ^neg^ CD56^dim^ NK Cells Develop Late and in Small Proportions in HCMV-Reactivating UCBT Recipients

**Figure 1:**
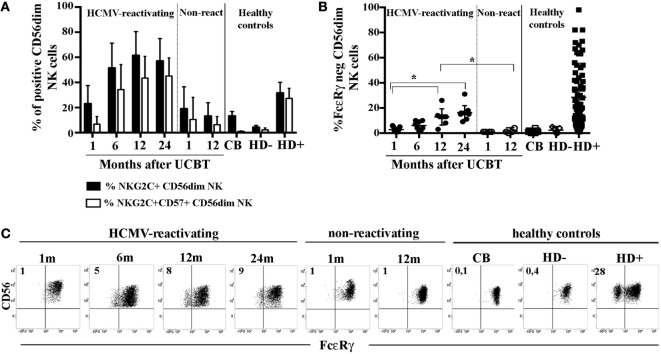
In human cytomegalovirus (HCMV)-reactivating umbilical cord blood transplantation (UCBT) recipients FcεRγ^neg^ CD56^dim^ natural killer (NK) cells develop late and in modest amounts. PB NK cells from the various patients were analyzed by multicolor immunofluorescence and FACS analysis at different time points after UCBT. After gating on CD56^dim^ NK cells, **(A)** the percentage of NKG2C^+^ NK cells (black bars) or NKG2C^+^CD57^+^ NK cells (white bars) were analyzed over time in both HCMV-reactivating (*n* = 9) and non-reactivating UCBT recipients (*n* = 5). For comparison, NK cells isolated from healthy controls, represented by cord blood (CB; *n* = 20), and adult healthy subjects, either HCMV seronegative (HD^−^; *n* = 10) or HCMV seropositive (HD^+^; *n* = 70), are shown. **(B)** FcεRγ expression was analyzed by intracellular immunofluorescence and FACS analyses in both patients and controls. The percentage of FcεRγ^neg^ CD56^dim^ NK cells was reported for each subject analyzed. 95%CI for the mean and statistical significance are indicated (*p < 0.05). **(C)** FcεRγ expression on CD56^dim^ NK cells is depicted at the various time points for one representative patient who experienced HCMV reactivation after UCBT and for another patient who did not. For comparison, CD56^dim^ NK cells from representative CB, HD^−^ and HD^+^ are reported. The percentage of FcεRγ^neg^ CD56^dim^ NK cells is indicated in the upper left quadrant.

In order to acquire insights in the generation of HCMV-induced, adaptive NK cells, we analyzed the FcεRγ expression in developing NK cells collected at different time points from a cohort of patients undergoing UCBT. In previous studies (23, 27), we showed that, in UCBT recipients experiencing HCMV reactivation (usually occurring within month 1–2 after transplantation), NK cell maturation was highly accelerated. In addition, a population of CD56^dim^ NK cells expressing NKG2C was greatly expanded and progressively acquired CD57, a marker typical of terminally differentiated NK cells. In the present study, we monitored the FcεRγ expression on CD56^dim^ NK cells. Despite the strong imprinting exerted by HCMV on reconstituting NK cells (as shown by the expansion of NKG2C^+^CD57^+^CD56^dim^ NK cells) (Figure 1A), adaptive FcεRγ^neg^ NK cells were either absent or barely detectable in most HCMV-reactivating patients up to 12 months after UCBT (Figure 1B). At this time point, FcεRγ^neg^ CD56^dim^ NK cells were detectable, although in lower proportions as compared to HCMV^+^ HD (indicated as HD^+^ in Figure 1B), while they were virtually undetectable both in UCBT recipients who did not experience HCMV reactivation (shown at 1 or 12 m) and in reactivating UCBT recipients early after HSCT (1–6 months). Notably, in HCMV-reactivating patients, the percentages of FcεRγ^neg^ CD56^dim^ NK cells were only slightly increased at 24 months after UCBT.

As expected, NK cells isolated from healthy controls including cord blood (CB) and HCMVseronegative healthy donors (HD^−^), did not display FcεRγ downregulation (Figure 1B). Notably, NK cells from HCMV seropositive healthy donors (HD^+^) were characterized by variable amounts of FcεRγ^neg^ cells (ranging from 2 to 95% with a median value of 20%, Figure 1B). On the other hand, in all cases, the adaptor protein CD3ζ was expressed by virtually all CD56^dim^ NK cells (data not shown). The CD56^bright^ NK cell subset consistently expressed both FcεRγ and CD3ζ, although at slightly lower levels than CD56^dim^ NK cells (data not shown). In Figure 1C, FcεRγ expression in CD56^dim^ NK cells is shown for two representative patients (one who did and the other who did not experience HCMV reactivation), in comparison to healthy controls either HCMV^−^ (CB and PB from HD^−^) or HCMV^+^ (PB from HD^+^).

Analysis of Syk expression revealed an expression pattern similar to that of FcεRγ, with the exception of two patients in whom Syk resulted partially downregulated already at early times after UCBT (data not shown).

### FcεRγ^neg^ NK Cells Emerging in HCMV-Reactivating UCBT Patients Mainly Belong to the NKG2C^−^CD57^+^ Subset and Only Partially to the Memory-Like NKG2C^+^ CD57^+^ Subset

**Figure 2:**
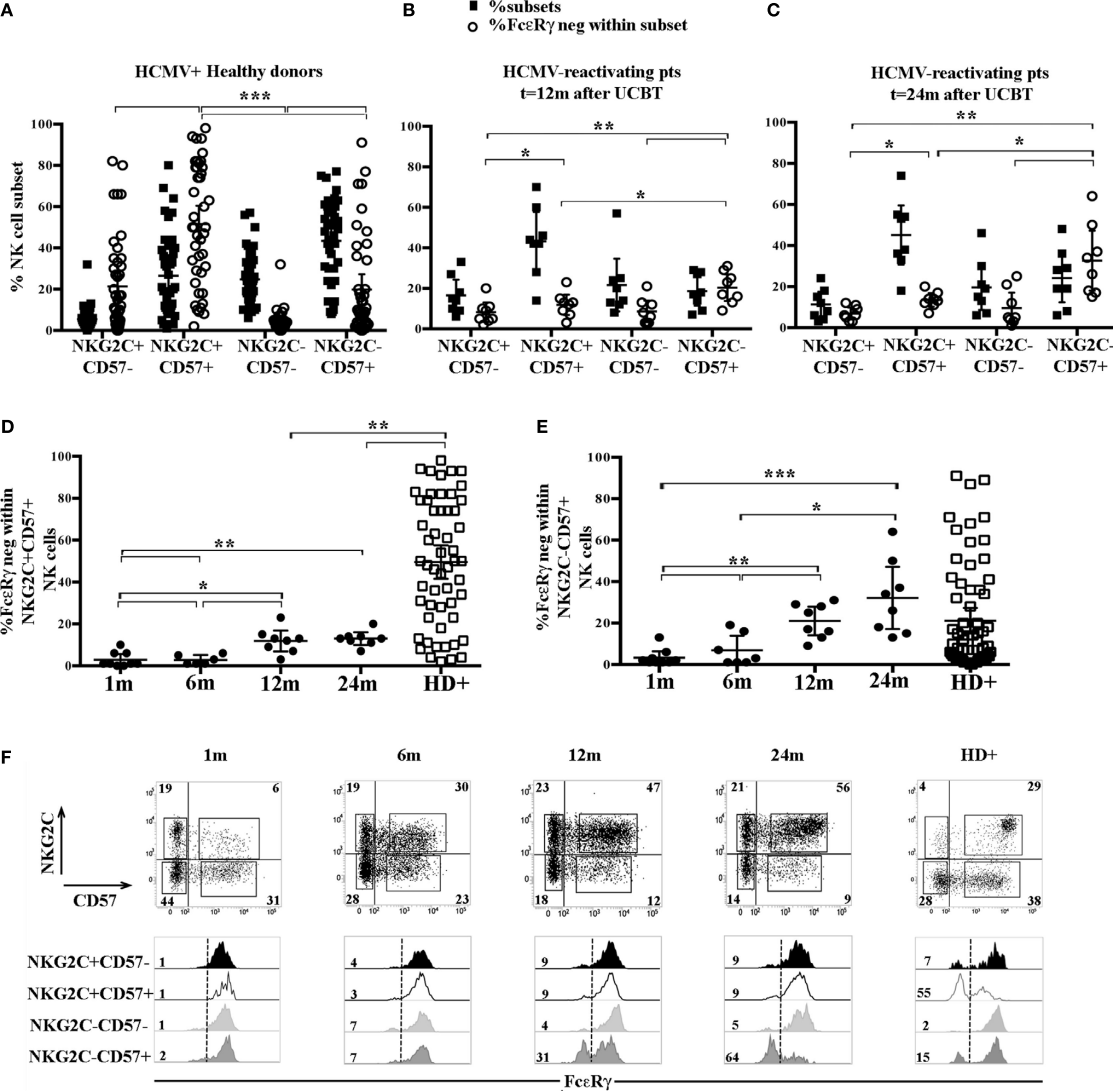
FcεRγ^neg^ natural killer (NK) cells emerging upon human cytomegalovirus (HCMV) reactivation in umbilical cord blood transplantation (UCBT) patients mainly belong to the NKG2C^−^CD57^+^ subset and in part to the classic memory-like NKG2C^+^ CD57^+^ subset. The distribution of the different CD56^dim^ NK cells subsets identified by NKG2C and CD57 expression was analyzed by multicolor immunofluorescence and FACS analysis in HCMV^+^ healthy donors (HD^+^) and HCMV-reactivating UCBT recipients. FcεRγ expression was subsequently evaluated in the different subsets identified by NKG2C/CD57 expression (i.e., NKG2C^+^CD57^−^, NKG2C^+^CD57^+^, NKG2C^−^CD57^−^, and NKG2C^−^CD57^+^). **(A–C)** Dot plots depict the frequency of the different NKG2C/CD57 subsets among CD56^dim^ NK cells (filled black squares), paralleled by the percentage of FcεRγ^neg^ NK cells within the indicated subset (empty white circles) in HCMV^+^ HD (**A**), HCMV-reactivating UCBT recipients at 12 (*n* = 8) **(B)** and 24 months (*n* = 8) **(C)** after transplant. **(D)** The percentage of FcεRγ^neg^ CD56^dim^ NK cells within the memory-like NKG2C^+^CD57^+^ NK cell subset is shown at the different time points in patients and in HCMV^+^HD for comparison. **(E)** The percentage of FcεRγ^neg^ CD56^dim^ NK cells within the NKG2C^−^CD57^+^ NK cell subset is shown at the different time points in UCBT recipients and in HCMV^+^ HD for comparison. 95%CI for the mean and statistical significance are reported (*p < 0.05; **p < 0.01; ***p < 0.001) in **(A–E)** panels. **(F)** NKG2C and CD57 expression is shown in CD56^dim^ NK cells from a representative HCMV-reactivating UCBT recipient over time (from 1 to 24 months). The percentages of positive cells are indicated in each quadrant. Below each dot plot, histogram plots representing FcεRγ expression in the different NKG2C/CD57 gated subsets are depicted as indicated on the left (black histograms: NKG2C^+^CD57^−^; white histograms: NKG2C^+^CD57^+^; light gray histograms: NKG2C^−^CD57^−^; dark gray histograms: NKG2C^−^CD57^+^). A representative HCMV^+^ HD is shown for comparison (HD^+^, right panels). Percentages of FcεRγ^neg^ NK cells are indicated for each subset.

It is well established that HCMV infection can promote, both in HD and in transplanted recipients, the expansion of a NKG2C^+^CD57^+^ CD56^dim^ NK cell subset possibly displaying adaptive properties (8, 23, 28–30). We further examined, in HCMV^+^HD, the FcεRγ expression analyzing four different cell subsets identified on the basis of the expression, or lack thereof, of NKG2C and CD57. This analysis revealed that downregulation/lack of FcεRγ expression was more frequent in the memory-like NKG2C^+^CD57^+^ CD56^dim^ NK cell subset. As shown in Figure 2A, where the frequency of each NKG2C/CD57 subset within CD56^dim^ NK cells is paralleled by the frequency of FcεRγ^neg^ cells within the corresponding subset, it can be seen that the NKG2C^+^ CD57^+^ subset displays a percentage of FcεRγ^neg^ cells significantly different from that of all other NKG2C/CD57 subsets. As previously described (31), FcεRγ^neg^ NK cells are virtually undetectable in the less differentiated NKG2C^−^CD57^−^ subset. On the other hand, FcεRγ^neg^ NK cells were detected in significant amounts not only in the memory-like subset but also in CD57^+^ cells lacking NKG2C (at least in some donors analyzed). This finding is in agreement with recent studies indicating that adaptive NK cells can develop also independently of NKG2C expression (16, 31, 32). In some donors, sizeable amounts of FcεRγ^neg^ cells were present also among NKG2C^+^CD57^−^ cells; however, this subset is poorly represented in HD (Figure 2A black squares). Along this line, we next examined the distribution of FcεRγ^neg^ cells in different CD56^dim^ NK cell subsets, as defined by NKG2C and CD57 expression from HCMV-reactivating UCBT recipients. In Figures 2B,C, the results for NK cells isolated at 12 and 24 months are shown. At these time points, FcεRγ^neg^ CD56dim NK cells became clearly detectable in UCBT recipients (Figure 1B). In line with HCMV^+^ HD, the highest frequencies of FcεRγ^neg^ NK cells were detected in both NKG2C^+^CD57^+^ and NKG2C^−^CD57^+^ subsets as compared to the other CD57^−^ subsets (Figure 2B, white circles). However, despite a strong HCMV-driven imprinting that favored a large expansion of the memory-like NKG2C^+^CD57^+^ cells (Figures 1A and 2B,C), FcεRγ^neg^ cells were preferentially present in the NKG2C^−^CD57^+^ subset. In fact, as shown in Figure 2D, the frequency of FcεRγ^neg^ cells among NKG2C^+^CD57^+^ cells, analyzed at the various time points, slowly increased over time, but remained significantly lower than that detected in HCMV^+^ HD even at 24 months after transplantation. On the contrary, the frequency of FcεRγ^neg^ cells among NKG2C^−^CD57^+^ NK cells increased over time and reached values similar or even slightly higher than those detected in HCMV^+^HD (Figure 2E). Small proportions of FcεRγ^neg^ cells were present also among the less mature NKG2C^−^CD57^−^ NK cells at 24 months (Figures 2B,C). In Figure 2F, FcεRγ expression in the different NKG2C/CD57 subsets is shown for a representative HCMV-reactivating patient in parallel to a representative HCMV^+^ HD.

**Figure 3:**
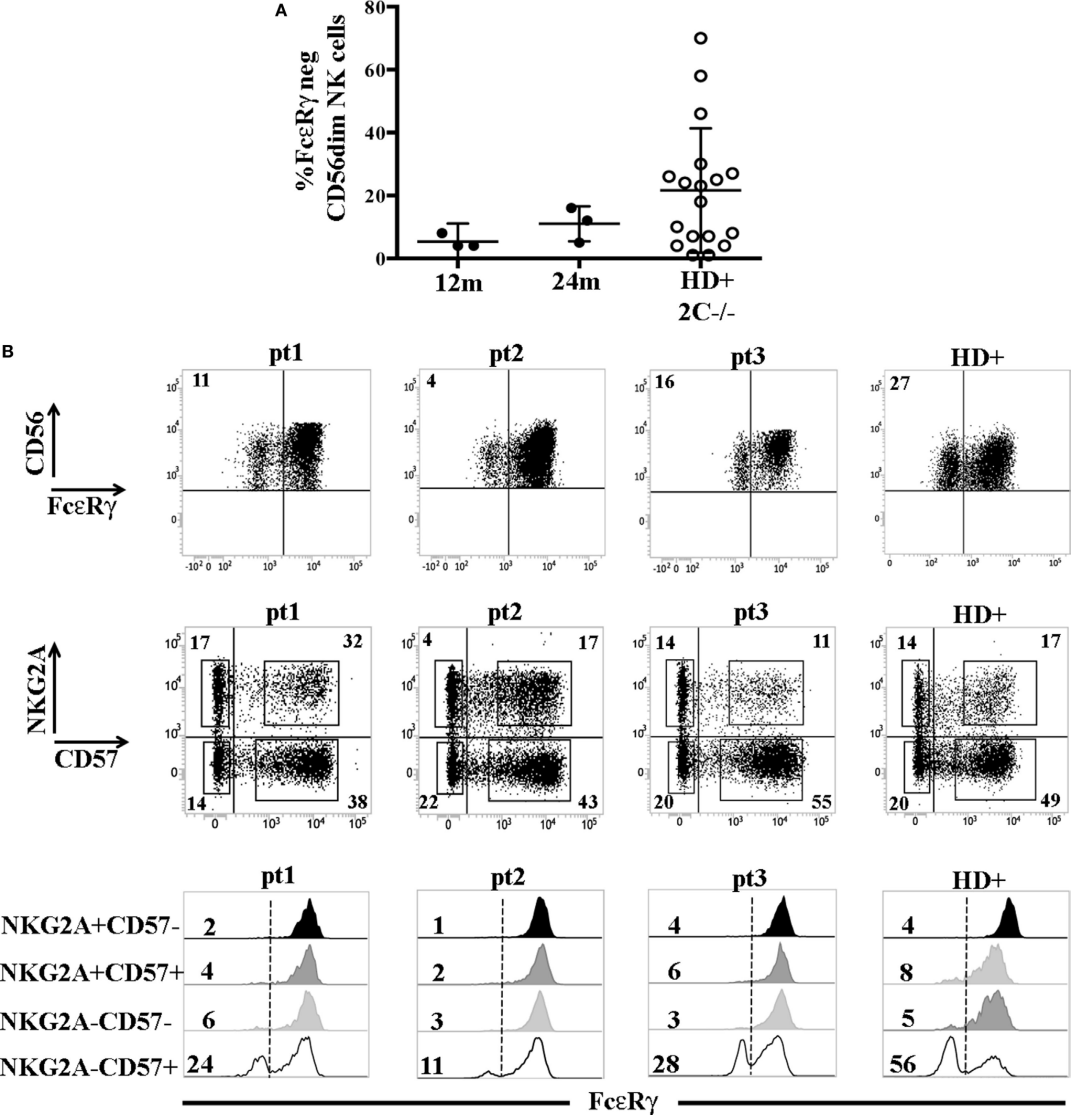
FcεRγ^neg^ natural killer (NK) cells are generated late also in human cytomegalovirus (HCMV)-reactivating recipients receiving umbilical cord blood transplantation (UCBT) from NKG2C^−/−^ donors. **(A)** The percentage of FcεRγ^neg^ CD56^dim^ NK cells in the three HCMV^−^ reactivating UCBT recipients who received a NKG2C^−/−^ donor is reported at 12 and 24 months after transplantation (FcεRγ^neg^ NK cells were virtually absent before 12 months). For comparison, the percentage of FcεRγ^neg^ CD56^dim^ NK cells found in HCMV^+^ healthy donors (HD) carrying the homozygous *NKG2C* gene deletion (HD^+^ 2 C^−/−^; *n* = 18) is indicated. 95%CI for the mean is reported. **(B)** In the upper panels, FcεRγ expression on CD56^dim^ NK cells is shown for the three patients at 24 months after UCBT and for a representative HCMV^+^ healthy donor NKG2C^−/−^ (HD^+^). The percentage of FcεRγ^neg^ CD56^dim^ NK cells is indicated in the upper left quadrant. Middle panels show reciprocal expression of NKG2A and CD57 in the different patients and in the representative HD^+^. The percentages of positive cells are indicated in each quadrant. In bottom panels, FcεRγ expression in the different NKG2A/CD57 gated subsets is depicted as indicated on the left (black histograms: NKG2A^+^CD57^−^; dark gray histograms: NKG2A^+^CD57^+^; light gray histograms: NKG2A^−^CD57^−^; white histograms: NKG2A^−^CD57^+^) for each patient and for HD^+^. The percentages of FcεRγ^neg^ NK cells are indicated for each subset.

The higher frequency of FcεRγ^neg^ NK cells in NKG2C^−^CD57^+^ NK cells could reflect the NKG2C copy number as suggested in a recent study (31) reporting that NKG2C^−^ FcεRγ^neg^ NK cells were more frequent in HCMV^+^ healthy individuals carrying only one copy (NKG2C^+/−^) as compared to those carrying two copies of the *NKG2C* gene (NKG2C*^+/+^*). We also compared NK cells developing in patients receiving UCBT from NKG2C^+/+^ or NKG2C^+/−^ donors, but we could not find appreciable differences in the frequencies of FcεRγ^neg^ cells both in NKG2C^+^CD57^+^ and in NKG2C^−^CD57^+^ NK cell subsets (Figure S2 in Supplementary Material). This suggests that, at variance with HD, in UCBT recipients, the generation of FcεRγ^neg^ cells in the NKG2C^−^CD57^+^ subset could be favored also by mechanisms independent from NKG2C copy number. Interestingly, in three HCMV-reactivating recipients receiving UCBT from NKG2C^−/−^ UCB donors (i.e., carrying homozygous *NKG2C* gene deletion), we detected a marginal FcεRγ downregulation at 12 months after the allograft that only slightly increased at 24 months (Figure 3A). In these patients, characterized by an expansion of mature (NKG2C^−^) NKG2A^−^KIR^+^CD57^+/−^ cells expressing aKIRs (27), FcεRγ^neg^ CD56^dim^ NK cells were preferentially found among terminally differentiated NKG2A^−^CD57^+^ NK cells (Figure 3B). This is in line with findings in HCMV^+^ HD carrying homozygous NKG2C deletion (Figure 3B right panels) (31, 32).

### NK Cells Developing in HCMV-Reactivating Patients After UCBT Acquire Several Adaptive Features Before FcεRγ Downregulation

**Figure 4:**
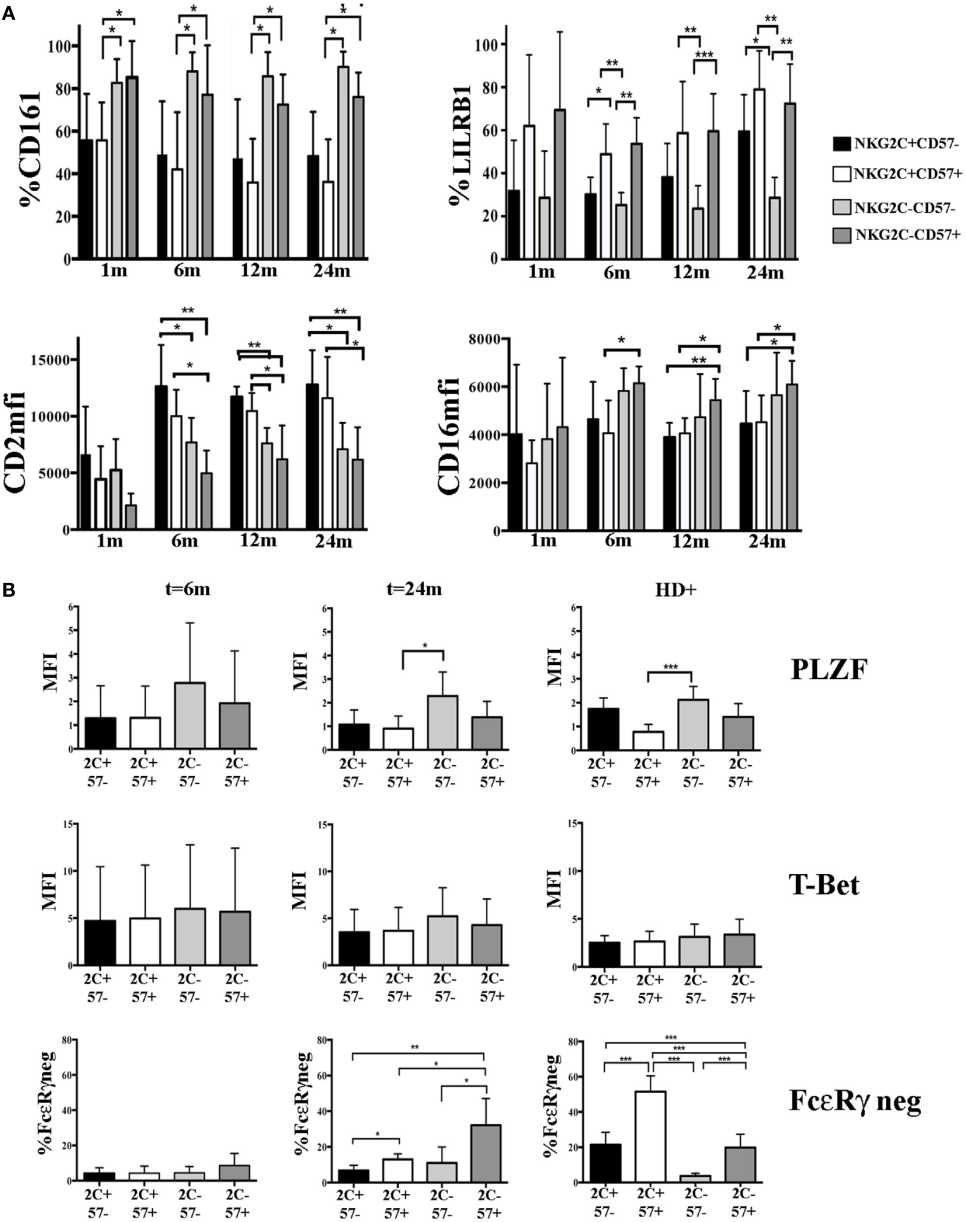
Modulation of surface receptors and transcription factors (TF) expression in NKG2C/CD57 natural killer (NK) cell subsets developing after umbilical cord blood transplantation (UCBT) in human cytomegalovirus (HCMV)-reactivating patients. PB-NK cells collected at different time points after UCBT from HCMV-reactivating patients and from HCMV^+^ healthy donors (HD^+^) were analyzed for the expression of the indicated surface markers, TF, and intracellular molecules, after gating on the different CD56^dim^ NK cell subsets identified by NKG2C and CD57 (i.e., black bars: NKG2C^+^CD57^−^; white bars: NKG2C^+^CD57^+^, light gray bars: NKG2C^−^CD57^−^, and dark gray bars: NKG2C^−^CD57^+^). **(A)** Upper panels show the percentage of CD161 (left) or LILRB1 (right) positive CD56^dim^ NK cells, while lower panels show CD2 (left) or CD16 (right) median fluorescence intensity (MFI), from month 1 to 24 after UCBT in the NKG2C/CD57 different subsets (*n* = 9). **(B)** The MFI of PLZF and T-Bet are shown at month 6 (*n* = 4) and 24 (*n* = 5) after UCBT in patients and in HD^+^ (*n* = 20) in the different NKG2C/CD57 subsets (indicated for brevity as 2C and 57). For comparison to TF expression, FcεRγ^neg^ CD56^dim^ NK cells are similarly depicted in the bottom panels. 95% CI for the mean percentage or MFI is shown and statistical significance is indicated (*p < 0.05; **p < 0.01; ***p < 0.001) in both **(A,B)**.

According to recent reports, HCMV-induced adaptive NK cells, besides the lack of FcεRγ expression, may also display a peculiar expression profile of given surface receptors which is likely to be determined by a specific DNA hypo- or hypermethylation pattern (16, 17). We analyzed some of these informative markers in CD56^dim^ NK cell subsets developing in HCMV-reactivating UCBT recipients. In parallel, we examined KIR and NKG2A expression to confirm the HCMV-driven skewing toward a mature KIR^+^NKG2A^−^ profile. As expected (23), higher precentages of KIR^+^NKG2A^−^ could be detected in NKG2C^+^ NK cells and in a fraction of NKG2C^−^CD57^+^ cells (Figure S3A in Supplementary Material). Interestingly, CD161 downregulation occurred mainly in NKG2C^+^ (both CD57^−^ and CD57^+^) NK cells as early as 1 month after UCBT, long before FcεRγ downregulation. CD161 expression was significantly lower in NKG2C^+^ than in NKG2C^−^ (both CD57^−^ and CD57^+^) NK cells at any time points (Figure 4A upper left panel). Of note, CD161 was homogeneously expressed by all NK cells isolated from non-reactivating recipients, CB and HCMV^−^HD (data not shown). Similarly, the upregulation of CD2 expression was higher in NKG2C^+^ NK cells (both CD57^+^ and CD57^−^) than in NKG2C^−^ cells already at 6 months in terms of MFI (Figure 4A bottom left panel), while no significant differences were found in terms of percentages of positive cells (Figure S3A in Supplementary Material). At variance with CD2 and CD161, the expression of LILRB1 was modified (upregulated) in both NKG2C^+^CD57^+^ and NKG2C^−^CD57^+^ NK cell populations and remained high over time (Figure 4A upper right panel). Finally, CD16 expression levels were significantly higher in NKG2C^−^CD57^+^ than in NKG2C^+^CD57^+^ cells by month 6 after UCBT (Figure 4A bottom right panel). As control, CD244 MFI was evaluated and did not show any significant difference among subsets (data not shown). These results are in line with data obtained in NK cells from HD experiencing HCMV infection (see Figure S3B in Supplementary Material).

Next, we investigated at early and late time points the expression of PLZF, a transcription factor involved in the regulation of epigenetic modifications that is either absent or reduced in adaptive NK cells (16). As shown in Figure 4B, we could detect a slight decrease in PLZF expression levels in NKG2C^+^CD57^+^ as compared to more immature NKG2C^−^CD57^−^ NK subset. While this was only a tendency at 6 months after UCBT, it subsequently increased becoming statistically significant at 24 months. Although, in the NKG2C^−^CD57^+^ subset, PLZF expression levels had a tendency to decrease, data were not statistically significant. NKG2C^+^CD57^−^ NK cells showed PLZF decrease both at 6 months and 24 months but without reaching statistical significance when compared to the other subsets. T-bet expression did not show significant differences among the various subsets. This was in line with what observed in HCMV^+^ HD, in whom PLZF downregulation mainly affected NKG2C^+^CD57^+^NK cells (Figure 4B right panels). It thus appears that after UCBT, HCMV can induce a potent reshaping of NK cell receptor repertoire long before FcεRγ downregulation. Such reconfiguration is characterized by a potent imprinting on NKG2C^+^CD57^+^ NK cells. Moreover, based on our observation, it is conceivable that PLZF dowregulation influenced the phenotypic reconfiguration prior to the FcεRγ downregulation (Figure 4B).

In line with the above observations indicating a rapid HCMV-induced phenotypic reconfiguration, followed by FcεRγ downregulation at late time points, we could not find any statistically significant difference between FcεRγ^+^ and FcεRγ^neg^ cell subsets at 24 months after UCBT (i.e., when FcεRγ^neg^ cells are measureable). However, NKG2C^−^CD57^+^FcεRγ^neg^ cells tended to be more differentiated than their FcεRγ^+^ counterpart (in terms of KIR and NKG2A expression). In addition, lower CD16 expression levels could be detected in FcεRγ^neg^ cells (Figures S4A–C in Supplementary Material). However, this difference was significant only in the NKG2C^+^CD57^+^ NK cell subset (Figure S4B in Supplementary Material). This is in agreement with data in HCMV^+^ HD (Figure S4B in Supplementary Material) and may be dependent on the requirement of FcεRγ for CD16 receptor expression (16, 21, 22).

### In HCMV-Reactivating UCBT Recipients FcεRγ Downregulation Occurs Earlier and in Larger Proportions in CD56^−^CD16^bright^ NK Subset Than in CD56^dim^ NK Cells

**Figure 5:**
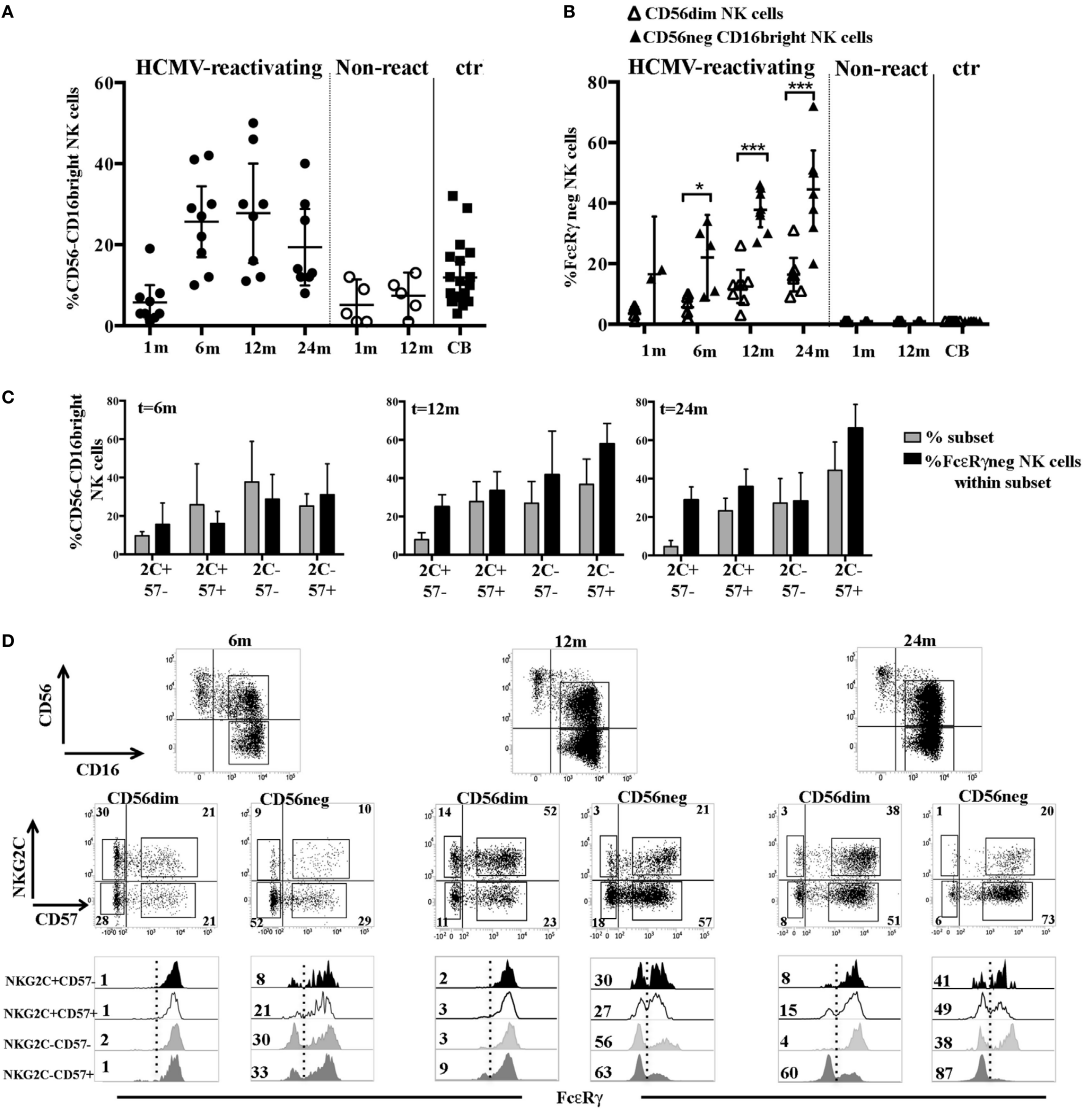
FcεRγ downregulation affects the CD56^−^CD16^bright^ natural killer (NK) cell subset earlier and to larger extent than CD56^dim^ NK cells in human cytomegalovirus (HCMV)-reactivating umbilical cord blood transplantation (UCBT) recipients. **(A)** The percentage of CD56^−^CD16^bright^ NK cells detected in both HCMV-reactivating (*n* = 9) (black circles) and non-reactivating (*n* = 5) (white circles) patients is shown at different time intervals after UCBT. NK cells isolated from cord blood (CB) are shown for comparison (black squares). 95% CI for the mean is indicated. **(B)** After gating on CD56^dim^ and CD56^−^CD16^bright^ NK cell subsets, the percentage of FcεRγ^neg^ NK cells was evaluated in these subsets and depicted as white (CD56^dim^) and black triangles (CD56^−^CD16^bright^), respectively, at the different time points in patients and in CB-NK cells. 95% CI for the mean and statistical significance is indicated (*p < 0.05; ***p < 0.001). **(C)** After gating on CD56^−^CD16^bright^ NK cells, FcεRγ expression was subsequently evaluated in the different NKG2C/CD57 subsets. Gray bars show the frequency of the indicated NKG2C/CD57 subset (reported for brevity as 2 C and 57), while black bars indicate the percentage of FcεRγ^neg^ NK cells evaluated within the same subset, in HCMV-reactivating UCBT recipients at 6, 12, and 24 months after transplantation. 95% CI for the mean is reported. **(D)** CD56 and CD16 expression on NK cells isolated from a representative patient characterized by the expansion of the CD56^−^CD16^bright^ NK cells subset is shown at 6, 12, and 24 months after UCBT. After gating on CD56^dim^ and CD56^−^CD16^bright^ NK cell subsets, reciprocal NKG2C and CD57 expression is shown over time for both subsets. The percentages of positive cells are indicated in each quadrant. Below each dot plot, histogram plots representing FcεRγ expression in the different NKG2C/CD57 gated subsets are depicted as indicated on the left (see also Figure 2C) for both CD56^dim^ and CD56^−^CD16^bright^ NK cell subsets. Percentages of FcεRγ^neg^ NK cells are indicated for each subset.

We previously demonstrated that in some UCBT recipients, upon HCMV infection/reactivation, a subset of hyporesponsive CD56^−^CD16^bright^ NK cells may appear by month 6 (23, 33) (Figure 5A). Unexpectedly, this subset displayed FcεRγ downregulation earlier (namely at 6 months) and to a larger extent as compared to CD56^dim^ NK cells from the same patients (Figure 5B). The lack of FcεRγ did not occur in CD56^−^CD16^bright^ NK cells isolated from CB, in which these cells can be easily detected (differently from adult PB where this subset is rare) (23, 34) (Figures 5A,B). Moreover, as shown in Figures 5C,D, CD56^−^CD16^bright^ FcεRγ^neg^ cells were differently distributed in NKG2C/CD57 subsets as compared to CD56^dim^ (Figures 2B,C). Interestingly, CD56^−^ FcεRγ^neg^ NK cells were found also in the NKG2C^−^CD57^−^ subset at any time point analyzed. The CD56^−^CD16^bright^ FcεRγ^neg^ NK cell subset was lineage negative (CD3, CD19, CD14, and CD33 negative) and expressed, in addition to CD3ζ (data not shown) and 2B4, high proportions of KIR and LILRB1 and low levels of NCRs and NKG2A (Figure S5A in Supplementary Material). Interestingly, in the patients developing the CD56^−^CD16^bright^ subset, the CD56^−^CD16^bright^ FcεRγ^neg^ cells dominated over CD56^dim^NKG2C^−^CD57^+^ FcεRγ^neg^ among total FcεRγ^neg^ cells at any time points (Figure S5B in Supplementary Material).

### In UCBT Recipients HCMV-Induced FcεRγ^neg^ and FcεRγ^+^ NK Cells Show Comparable Functional and Proliferative Capabilities

**Figure 6:**
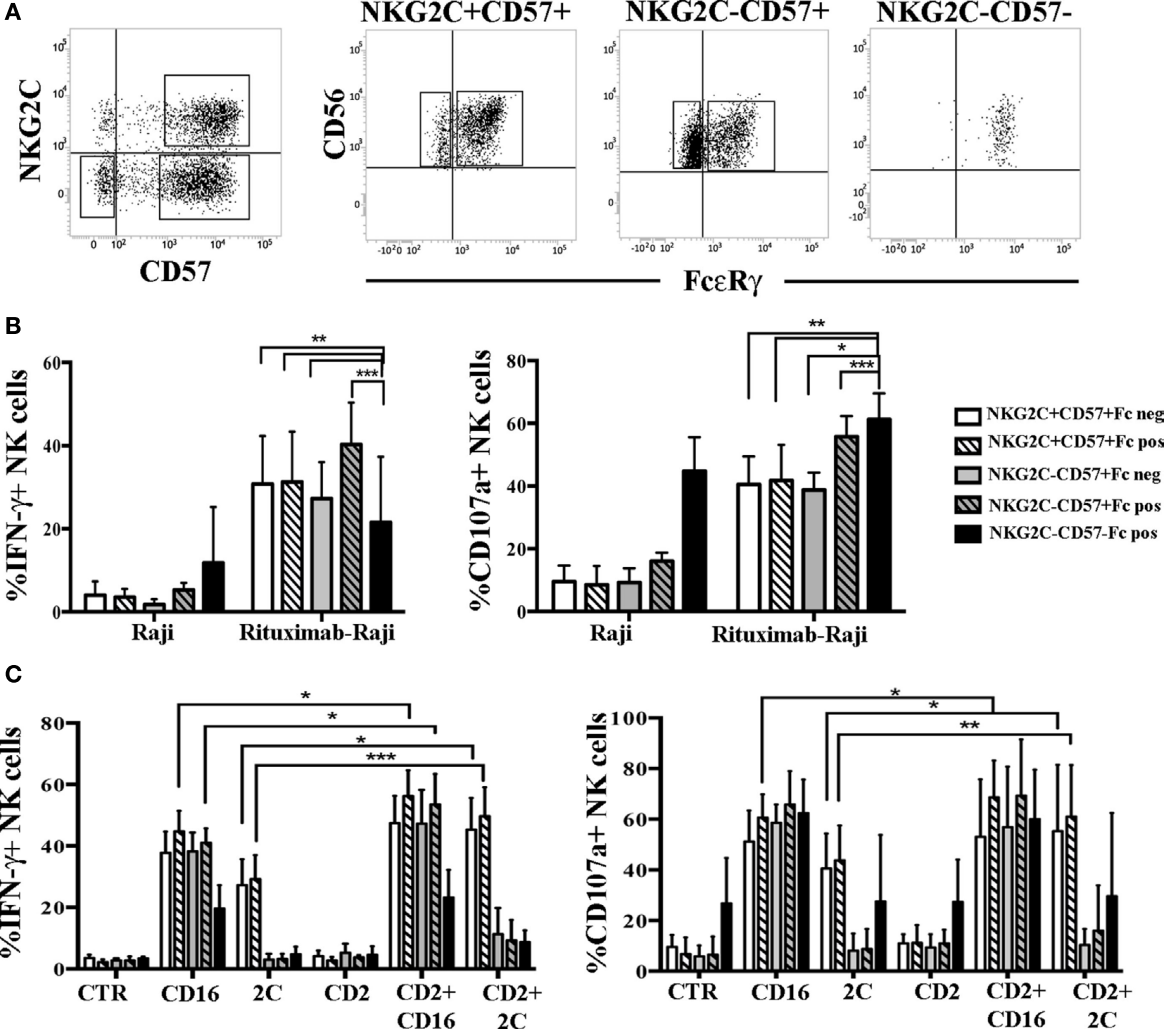
Human cytomegalovirus (HCMV)-induced FcεRγ^neg^ an FcεRγ^+^ natural killer (NK) cell subsets show comparable IFN-γ production and degranulation capabilities. **(A)** The gating strategy used to identify the different NK cells subsets analyzed in functional assays is shown for a representative patient at 24 months after umbilical cord blood transplantation (UCBT). After gating on CD56^dim^ NK cells, NKG2C^+^CD57^+^ and NKG2C^−^CD57^+^ NK cell subsets were identified and subsequently gated according to FcεRγ (Fc for brevity) expression in NKG2C^+^CD57^+^FcεRγ^+^ or NKG2C^+^CD57^+^FcεRγ^neg^ and in NKG2C^+^CD57^+^FcεRγ^+^ or NKG2C^+^CD57^+^FcεRγ^neg^, respectively. The NKG2C^−^CD57^−^ NK cells, which are virtually all FcεRγ^+^, were analyzed in parallel. **(B)** Peripheral blood mononuclear cell (PBMC) from HCMV-reactivating patients (*n* = 6), collected at 24 months after UCBT, were cultured overnight in the presence of rhIL-15. PBMC were then washed and cultured in the presence or in the absence of the Raji cell line, previously coated or not with the human anti-CD20 Rituximab (1 µg/mL), at an E:T ratio of 1:1, for 4 h. Thereafter, IFN-γ production (left panel) and CD107a degranulation (right panel) were evaluated for the indicated NK cells subsets described in the figure. PBMC alone were virtually IFN-γ and CD107a negative in all subsets and were not depicted. 95%CI for the mean of CD107a/IFN-γ positive NK cells is shown for each subset. Statistical significance is indicated (*p < 0.05; **p < 0.01; ***p < 0.001). **(C)** After overnight culture, the same cells were incubated with the FcγR^+^ murine p815 cell line either in the presence or in the absence of anti-CD16, anti-NKG2C (indicated as 2C), anti-CD2 monoclonal antibodies (mAbs) alone or in combination. CTR indicates cells cultured in the presence of p815 and in the absence of mAbs.

Previous studies suggested that the lack of FcεRγ could favor a more efficient NK cell-mediated response to Ab-coated targets (17, 35). Thus, we compared degranulation and IFN-γ production in FcεRγ^neg^ and FcεRγ^+^ NK cells belonging to the memory NKG2C^+^CD57^+^ or to the NKG2C^−^CD57^+^ cell subsets against Rituximab-coated Raji cell line (see Figure 6A for gating strategy). In this set of experiments, NK cells isolated at 24 months after transplantation from HCMV-reactivating patients were analyzed. As shown in Figure 6B, FcεRγ^neg^ and FcεRγ^+^ NK cells were equally effective in terms of IFN-γ production, despite the fact that NKG2C^+^CD57^+^FcεRγ^neg^ cells expressed CD16 at lower levels (Figure S4B in Supplementary Material). On the other hand, the response to Rituximab-coated Raji of both NKG2C^+^CD57^+^ and NKG2C^−^CD57^+^ NK cells were higher as compared to the less differentiated NKG2C^−^CD57^−^FcεRγ^+^ NK cell subset. In parallel experiments, the level of CD107a degranulation against Rituximab-coated Raji cells was comparable in FcεRγ^neg^ and FcεRγ^+^ NK cells from both CD57^+^ subsets. However, in this case, the NKG2C^−^CD57^−^ cells strongly responded to uncoated Raji, thus making difficult to compare their response to mAb-coated Raji to that elicited by CD57^+^ cells (Figure 6B). This high degranulation ability is likely to reflect a higher expression of NKp30 [which is primarily involved in the recognition of the B7-H6^+^ Raji cell line (36)], typical of less differentiated NK cells, as compared to CD57^+^ cells (data not shown) (37, 38). Similar data were obtained in HCMV^+^ HD (HD^+^) (see Figure S6A in Supplementary Material).

Based on recent studies (32), in HD^+^ adaptive FcεRγ^neg^ NK cells displayed enhanced responses to CD16-mediated triggering when the CD2 co-receptor was simultaneously engaged. Notably, in the rituximab-coated-Raji assays such co-stimulation can always occur as Raji cells express CD58, the CD2 ligand, thus making the CD2-mediated increases not easily appreciable. Thus, we performed a more appropriate mAb-mediated crosslinking assay to better investigate the possible role of CD2 coengagement in the functional response mediated by FcεRγ^neg^ and FcεRγ^+^ NK cells derived from UCBT patients. As shown in Figure 6C, when CD16 and CD2 were simultaneously engaged by mAb-mediated triggering, we could detect a modest increase in IFN-γ production and CD107a degranulation as compared to CD16 stimulation alone. This increase was significant only in NKG2C^+^CD57^+^FcεRγ^+^ NK cells (but not in NKG2C^+^CD57^+^FcεRγ^neg^ NK cells). The level of increase paralleled the CD2 expression levels in both FcεRγ^+^ and FcεRγ^neg^ subsets (Figure S4B in Supplementary Material). Interestingly, CD2 co-stimulation was even more effective in augmenting both IFN-γ production and degranulation induced by NKG2C triggering in NKG2C^+^CD57^+^ NK cells (both FcεRγ^+^ and FcεRγ^neg^) (Figure 6C). However, CD2 co-stimulation could increase CD16-triggered responses also in the NKG2C^−^CD57^+^ subset (both FcεRγ^+^ and FcεRγ^neg^) although a modest significance was reached only in NKG2C^−^CD57^+^ FcεRγ^pos^ cells (Figure 6C), although CD2 expression levels were lower than those found in NKG2C^+^ NK cells (Figure 4; Figure S4B in Supplementary Material). Similar data were obtained in HD^+^ (see Figure S6B in Supplementary Material).

In conclusion, we could not observe substantial differences in the effector function characterizing FcεRγ^+^ and FcεRγ^neg^ cells both in the memory-like NKG2C^+^CD57^+^ and in the NKG2C^−^CD57^+^ subset. This may indicate that full effector function and adaptive features (such as CD2-mediated enhancement of CD16 and NKG2C responses) could be achieved by HCMV-induced NK cells before and independently from FcεRγ downregulation after UCBT.

**Figure 7:**
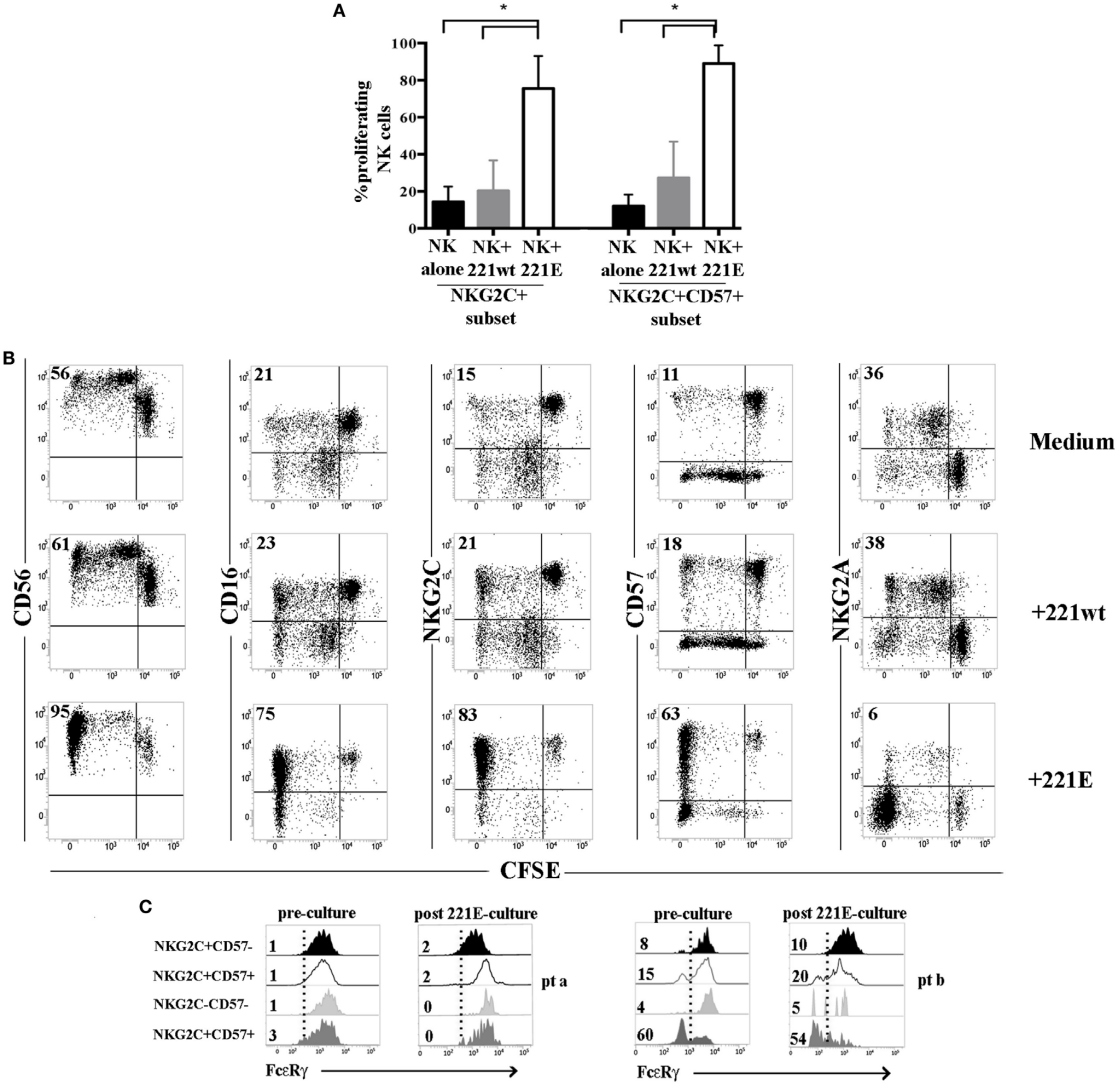
NKG2C^+^ (CD57^+^) natural killer (NK) cells from human cytomegalovirus (HCMV)-reactivating patients proliferate efficiently in response to NKG2C stimulation independently from FcεRγ expression. Peripheral blood mononuclear cells from HCMV-reactivating patients (*n* = 5) collected at 24 months after umbilical cord blood transplantation (UCBT) were CFSE-labeled and cultured either in the presence or in the absence of 221wt or 221AEH (indicated as 221E). FACS analyses on NK cells were performed as described at 7, 10, and 14 days of coculture, by gating on CD56^+^ CD3^−^CD19^−^CD14^−^ lymphocytes. **(A)** The percentage of proliferating (i.e., undergoing CFSE dilution) NKG2C^+^ or NKG2C^+^CD57^+^ NK cells in the different culture conditions are depicted (black bars: NK alone; gray bars: NK + 221wt; white bars: NK + 221E) at day 10 of coculture 95%CI for the mean and statistical significance is indicated (*p < 0.05). **(B)** The expression of the the indicated markers is shown on NK cells from a representative HCMV-reactivating UCBT patient in the different culture conditions at day 10. NK cell proliferation is determined by CFSE dilution. Percentages of proliferating NK cells are indicated in the upper left quadrant. **(C)** FcεRγ expression is shown for the different NKG2C/CD57 NK cell subsets, identified as described above, in two representative patients comparing results obtained before (preculture) and after 14 days of coculture in the presence of 221E (postculture). Percentages of FcεRγ^neg^ NK cells are indicated for each subset.

We next investigated whether, in UCBT recipients, adaptive NKG2C^+^CD57^+^ FcεRγ^neg^ NK cells differentiating at late time points could be expanded upon exposure to a transfected cell line (721.221AEH, 221E for brevity) expressing the specific NKG2C ligand HLA-E (10, 39). In CFSE proliferation assays, after 7–10 days coculture, we could detect a remarkable expansion of NKG2C^+^CD57^+^(NKG2A^−^) NK cells exposed to the HLA-E^+^ cell line (221E). Controls included NK cells cultured either alone or in the presence of the non-transfected (221wt) cell line (Figures 7A,B). In line with previous reports (40–42), we could observe similar responses in HD^+^ (Figure S7 in Supplementary Material). Remarkably, after 221E-induced proliferation, NK cells maintained the same percentages of FcεRγ^neg^ cells present in resting NK cells (pre-culture) in both patients and HD^+^ (Figure 7C; Figure S7 in Supplementary Material), suggesting that both subsets (NKG2C^+^CD57^+^FcεRγ^+^ and NKG2C^+^CD57^+^FcεRγ^neg^) could efficiently proliferate in response to stimuli acting on the NKG2C receptor.

## Discussion

In the context of HCMV infection, human NK cells were shown to display unexpected adaptive traits, sharing features typical of T cells, including enhanced effector function, oligoclonal expansion, and long-term survival in response to a viral challenge (17, 23, 28, 33, 41, 43). Although a precise phenotypic definition of adaptive NK cells cannot be provided, HCMV-induced expanded NK cells are mostly represented by terminally differentiated NKG2C^+^CD57^+^selfKIR^+^ NKG2A^−^ NK cells (23, 24, 28, 33). Importantly, according to recent studies in HCMV^+^ HD, HCMV-induced NK cells display adaptive features including a decreased expression of signaling molecules (FcεRγ, SYK, EAT-2, DAB) and transcription factors (PLZF). Moreover, these cells can undergo epigenetic modifications that lead to changes in their phenotype (e.g., downregulated expression of CD161, CD7, Siglec-7, IL-12R, and IL-18R and upregulated expression of CD2 and LILRB1) and function (e.g., more efficient ADCC, lower response to IL-12 and IL-18) (16, 17).

Notably, in view of the functional features and longevity of adaptive NK cells, they represent suitable candidates to be exploited in cellular immunotherapy against cancer. However, a better understanding of the mechanisms involved in the generation of this NK cell subset is necessary. With the purpose of clarifying these mechanisms and establishing at which stage the described adaptive features are acquired by NK cells upon HCMV encounter, we carried out a study in a cohort of patients undergoing UCBT. In this transplantation setting, HCMV infection/reactivation is known to drive a strong NK cell skewing toward phenotypically and functionally polarized cell populations (23, 24). Notably, NK cells are the first lymphocyte population which reconstitutes after transplantation and, thus, they may receive a strong imprinting by HCMV, especially in transplantation settings in which T cell immunity remains impaired for a prolonged period of time (44). In this study, we show that certain adaptive characteristics are acquired early in UCBT recipients experiencing HCMV reactivation (within 6 months after UCBT), while others appear later and are confined to a small fraction of developing NK cells. In particular, our data indicate that HCMV induced a rapid reconfiguration of the surface phenotype, by promoting the expansion of NKG2C^+^CD57^+^ NK cells, mainly NKG2A^−^KIR^+^, displaying high CD2 and LILRB1 and low CD161 expression by month 6. On the other hand, downregulation of the signaling adaptor protein FcεRγ, which represents a crucial adaptive feature in NK cells of HCMV^+^ HD, appeared only late, namely at 12 months after UCBT and remained confined to a small cell subset even at 24 months. Notably, FcεRγ downregulation could be detected at higher frequency in CD57^+^ NK cells lacking NKG2C than in the classic memory-like NKG2C^+^CD57^+^ subset, despite the fact that this subset was the most expanded one and displayed an evident HCMV-induced reconfiguration (Figures 2B,C and 4). Adaptive FcεRγ^neg^, NK cells lacking NKG2C have been previously described (31, 32, 43). Accordingly, we found variable proportions of this NK cell subset in HCMV^+^ HD(Figure 2A). A less skewed signature compared to the NKG2C^+^CD57^+^ NK cell subset characterized this subset, although it showed a tendency to be more mature than its FcεRγ^+^ counterpart, especially in HD^+^ (Figures S4B,C in Supplementary Material). In order to determine whether this CD57^+^NKG2C^−^ FcεRγ^neg^ subset is persistent in transplanted patients, further assessments at later time intervals after UCBT would be necessary.

Interestingly, we detected a significant downregulation of the transcription factor PLZF in the memory-like NKG2C^+^CD57^+^ NK cell subset (both FcεRγ^pos^ and FcεRγ^neg^) at 24 months after UCBT, while, as shown above, FcεRγ downregulation remained confined to a small subset (Figures 2 and 4B; Figure S5 in Supplementary Material). This expression pattern differed from HCMV^+^ HD, in which, in line with previous reports (16), the majority of NKG2C^+^CD57^+^ NK cells displayed both PLZF and FcεRγ downregulation (Figure 4B). It cannot be ruled out that the PLFZ downregulation may precede the FcεRγ one in memory-like cells, contributing to the early reconfiguration of the NKG2C^+^ cell subset. Indeed, PLZF can act as a transcriptional repressor, but can also induce gene expression. Thus, its absence may favor the acquisition of some adaptive features, for example by determining the low expression of IL-18R, IL-12R, and CD161 (16, 45). Moreover, its absence may confer long-living properties, as suggested by data on MAIT cells, in which PLZF represents a pro-apoptotic factor (46). On the other hand, in UCBT recipients, the two phenomena (PLZF and FcεRγ downregulation) occurred independently in different cells, since FcεRγ downregulation was detected in NKG2C^−^CD57^+^ NK cells that do not display a discrete PLZF downregulation at 24 months after transplantation (Figure 4B; Figure S5 in Supplementary Material).

It is possible that the partial differentiation of adaptive NK cells lacking FcεRγ downregulation could be related to the fact that repeated HCMV infection/reactivation episodes may be required to reach a stable epigenetic reprogramming, capable of silencing FcεRγ (16). It is also possible that other viral infections (and/or vaccines) different from HCMV, frequently occurring in HD over the lifespan, may also play a role in shaping adaptive NK cell differentiation. Along this line, the generation of adaptive FcεRγ^neg^ NK cells may require a network of signals and cellular interactions (e.g., cytokines or other soluble factors released at inflammatory sites, and/or interactions with infected targets, and/or humoral immunity responses) that could be temporarily altered in UCBT recipients in whom the acquisition of adaptive immunity occurs late. In this context, the presence of expanded adaptive NKG2C^+^ NK cells that had not downregulated FcεRγ has been found also in TAP-deficient patients (47).

Additional findings suggest a role for persistent viral stimulations in generating FcεRγ^neg^ NK cells. Thus, in some transplanted patients characterized by the emergence of CD56^−^CD16^bright^ NK cells following HCMV reactivation (23), we found that this subset displayed an early downregulation of FcεRγ occurring independent of NKG2C and increasing over time (Figure 5). Since CD56^−^CD16^bright^ NK cells are likely to be generated in response to chronic immune activation (48, 49), we can infer that persistent stimulation could also act as an inducer of FcεRγ downregulation. In line with this hypothesis, CD56^−^16^bright^ FcεRγ^neg^ NK cells have been described in chronically infected HCV patients (50). The patients examined in that study were HCMV^+^, but did not reactivate HCMV (no viral DNA was detected), suggesting a possible role for HCV infection in the generation of FcεRγ^neg^ NK cells. Interestingly, similar expansions of CD56^−^16^bright^ FcεRγ^neg^ NK cells were found also in some HCMV^+^GATA2-deficient patients (43). Finally, it is possible that, following other stimuli or cytokine exposure, these CD56^−^ FcεRγ^neg^ cells may recover CD56 expression, while maintaining FcεRγ downregulation. However, further studies are clearly necessary to unravel the origin and function of this peculiar subset.

In line with the hypothesis that multiple or persistent signals are needed to fully differentiate such adaptive NK cell subsets, when NKG2C^+^CD57^+^ NK cells from UCBT patients were exposed to 221.AEH expressing the NKG2C ligand HLA-E, we could detect a prompt proliferative response of this subset independent of the expression of FcεRγ (Figure 7). It is likely that the experimental system based on NKG2C stimulation through HLA-E^+^ targets is not sufficient to provide stimuli capable of inducing either FcεRγ downregulation or a preferential expansion of FcεRγ^neg^ cells. Indeed, according to other studies, FcεRγ^neg^ NK cell proliferation is favored in the presence of IgG Abs engaging CD16 (16, 17). Thus, we cannot exclude that the low percentage of FcεRγ^neg^ NK cells may depend on compromised humoral responses in our UCBT recipients.

Another important issue concerns the possibility that adaptive NK cells may play a beneficial role in anti-leukemic responses after transplantation (33, 51). Remarkably, FcεRγ^neg^ adaptive NK cells have been described in HD to display a strong ADCC activity that can be further increased by CD2-mediated co-stimulation (32). In our study, we could not detect relevant differences in the effector function (i.e., IFN-γ production and degranulation) of FcεRγ^+^ and FcεRγ^neg^ cells belonging either to NKG2C^+^CD57^+^ or to the NKG2C^−^CD57^+^ subset. This suggests that, after UCBT, full effector function and adaptive features may be achieved by HCMV-induced NK cells before and/or independently of FcεRγ downregulation. The finding that FcεRγ^+^ and FcεRγ^neg^ NK cells display similar functional capabilities would be in partial disagreement with previous reports in HD (17). However, this difference could also be related to different gating strategies. Thus, in our study, we analyzed the features of FcεRγ^neg^ NK cells within the classic NKG2C^+^CD57^+^ memory-like subset and the NKG2C^−^CD57^+^ subset. This strategy may lead to results which differ, at least in part, from those comparing FcεRγ^neg^ and FcεRγ^+^ NK cells. Along this line, Muntasell et al. showed that in HD^+^ NKG2C^+^FcεRγ^+^ and NKG2C^+^FcεRγ^neg^ NK cells responded similarly in terms of TNF-α release upon stimulation with Rituximab-coated targets (31) and suggested that NKG2C expression and FcεRγ loss represented uncoupled events along the acquisition of the HCMV-induced signature and functionality (31). Moreover, Moraru et al. (52) showed that NKG2C^bright^CD57^+^ FcεRγ^neg^ and FcεRγ^+^ subsets displayed similar degranulation capabilities against opsonized HSV-infected targets. It should also be considered that a balance of opposing effects could occur in the NKG2C^+^CD57^+^ FcεRγ^neg^ subset that is characterized by lower CD16 expression levels as compared to the NKG2C^+^CD57^+^FcεRγ^+^ cell subset (Figure S3C in Supplementary Material). However, the lower CD16 expression could be compensated by a stronger signal delivered by the adaptor signaling protein CD3ζ (containing three ITAMs) as compared to a weaker signal delivered by CD16 when it is coupled to FcεRγ (containing only 1 ITAM) (21, 22).

Epigenetic and proteomic studies on larger cohorts of patients receiving UCBT are necessary for further unraveling the mechanisms behind adaptive NK cell generation, expansion, and longevity. However, our study highlights that, in UCBT recipients, HCMV reactivation may induce a rapid phenotypic reconfiguration including an early acquisition of certain adaptive features, while FcεRγ downregulation occurs only at later stages. Thus, while in these patients the complete acquisition of adaptive, HCMV-induced features by developing NK cells requires long time intervals, the rapid HCMV-driven reconfiguration is accompanied by the acquisition and persistence of the effector functions. This finding could be applied to design novel immunotherapeutic strategies (e.g., CAR-NK cells generation by manipulation of HCMV-induced long-living NK cell subsets) exploiting the functional potential of different subsets of adaptive NK cells, such as those emerging in UCBT recipients experiencing HCMV reactivation.

## Ethics Statement

Either patients or their parents gave their informed consent to participation in this study, which was approved by the Azienda Ospedaliera Universitaria San Martino (Genoa, Italy), by the University of Genoa and by the Bambino Gesù Children’s Hospital (Rome, Italy) ethics committees and was conducted in accordance with the tenets of the Declaration of Helsinki.

## Author Contributions

LMuccio designed research, performed experiments, and contributed to paper writing; MF performed experiments and contributed to data analysis and paper writing; AB recruited study subjects and contributed to data analysis and paper writing; FF recruited study subjects and critically revised the paper; SS critically revised the paper and provided economic support; FL and LMoretta interpreted data and critically revised the paper; AM interpreted data, provided economic support, and wrote the paper; MDC designed research, performed experiments, interpreted data, and wrote the paper.

## Conflict of Interest Statement

AM is founder and shareholder of Innate-Pharma (Marseille, France). The remaining authors have no conflicting financial interests to disclose.
