# Supplementary material for: Late Development of FcεRγneg Adaptive Natural Killer Cells Upon Human Cytomegalovirus Reactivation in Umbilical Cord Blood Transplantation Recipients
Source: Front Immunol. 2018 May 15;9:1050. doi: 10.3389/fimmu.2018.01050 (PMC5968376; doi:10.3389/fimmu.2018.01050)
Supplement: Supplementary file 1 [file data_sheet_1.PDF]

Supplementary Material

Late development of FcεRγ^{neg} adaptive NK cells upon HCMV reactivation in UCBT recipients

Letizia Muccio, Michela Falco, Alice Bertaina, Franco Locatelli, Francesco Frassoni, Simona Sivori*, Lorenzo Moretta, Alessandro Moretta° and Mariella Della Chiesa*°

Corresponding Authors: Mariella Della Chiesa; e-mail: mariella.dellachiesa@unige.it and Simona Sivori; simona.sivori@unige.it

Table s1

Pt #	Age	Gender	Diagnosis	Conditioning regimen	HCMV infection/ reactivation	HCMV serostatus D/R	Day after UCBT when HCMV infection/ reactivation occurred	Multiple episodes of HCMV infection/ reactivation	Other viral infections	GVHD, grade	n°CD34^{+} cells infused(x 10^5)
1	2 y	F	AML	Bu/Cy/Mel	no	neg/neg	na	na	no	no	2,34
2	11 m	M	MPS type I	Bu/TT/Flu	no	neg/neg	na	na	no	yes, acute III	3,26
3	7 m	M	Omenn S.	Treo/TT/Flu	no	neg/neg	na	na	no	yes, acute IV	3,36
4	5 y	F	ALL	TBI/TT/Flu	yes	neg/pos	35	yes	HHV-6	no	3,75
5	10 y	M	AML	Bu/Cy/Mel	yes	neg/pos	24	yes	BK	yes, acute III	1,89
6	7 y	M	ALL	TBI/TT/Flu	yes	neg/pos	27	yes	no	yes, acute III	2,2
7	2 y	F	AML	Bu/Cy	yes	neg/pos	19	no	no	yes, acute II	2,1
8	33 y	F	AML	CY+TBI	no	neg/neg	na	na	no	yes, acute 1	1.89
9	46 y	F	AML	CY+TBI	no	neg/neg	na	na	EBV	yes, acute and chronic I	2.7
10	21 y	M	AML	CY+TBI	yes	neg/neg	24	yes	no	yes, chronic I	2.16
11	44 y	M	AML	CY+TBI	yes	neg/pos	23	yes	no	yes, acute I	1.7
12	35 y	M	MDS	CY+TBI	yes	neg/pos	67	yes	no	no	1.0
13	47 y	F	AML	CY+TBI	yes	neg/pos	65	no	EBV	no	2.75
14	38 y	F	AML	CY+TBI	yes	neg/pos	35	no	no	no	4.1
15	63 y	M	CML	THIO-TREO-FLU	yes	neg/pos	55	yes	no	yes, acute and chronic I	0.68
16	34 y	F	AML	CY+TBI	yes	neg/pos	60	yes	no	yes, acute 11	1.1
17	39 y	M	AML	CY+TBI	yes	neg/pos	42	yes	EBV	no	3.4

Table legend: AML, acute myeloid leukemia; MPS, mucopolysaccharidosis; ALL, acute lymphoblastic leukemia; MDS, myelodysplastic syndrome; CML, chronic myeloid leukemia; Bu, busulfan; TT, Thiotepa; FLU, Fludarabine; TREO, Treosulfan; TBI, Total Body Irradiation; Mel, melphalan; Cy, cyclophosphamide; D/R, donor/recipient; UCBT, umbilical cord blood cell transplantation; HCMV, human cytomegalovirus; HHV6, Human Herpesvirus 6; EBV, Epstein-Barr virus; GvHD, Graft-versus-Host Disease; na, not applicable.

Supplementary Figures

Figure s1

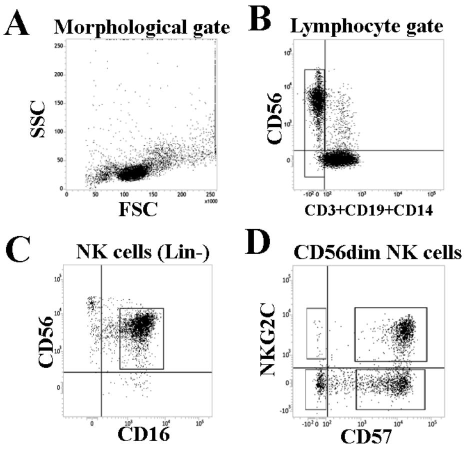

Figure s1. Gating strategy. PBMC from a representative HCMV^{+} healthy donor is shown. NK cells were gated first as lymphocytes by physical parameters (**A**) and then identified by the combined use of anti-CD56 vs anti-CD3, anti-CD19 and anti-CD14 mAbs as CD3^{-}CD19^{-}CD14^{-} (Lin^{-}) CD56$^{+/-}$ cells (**B**). Lin^{-} NK cells were then divided in CD56^{bright}, CD56^{dim} and CD56^{neg} subsets according to the reciprocal expression of anti-CD56 and anti-CD16 mAbs (**C**). The CD56^{dim} NK cell subset was subsequently labeled with anti-NKG2C and anti-CD57 mAbs to identify the different NKG2C/CD57 CD56^{dim} subsets to be analyzed for phenotype and function (**D**). CD56^{neg} NK cells were similarly gated in NKG2C/CD57 subsets where indicated.

Figure s2

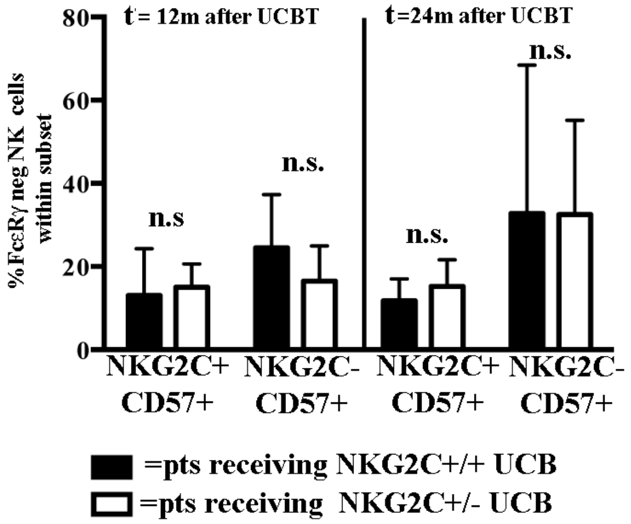

Figure s2. NKG2C gene copy number does not influence FcεRγ downregulation. The percentages of NKG2C$^+$CD57$^+$FcεRγ^{neg} NK cells and NKG2C$^-$CD57$^+$ FcεRγ^{neg} have been reported in patients who received UCBT either from NKG2C$^{+/+}$ UCB donors (n=4, black bars) or from NKG2C$^{+/-}$ UCB donors (n=4, white bars). Data obtained from PBMC collected at 12 months and 24 months after UCBT are shown on the left, and right part of the figure, respectively. 95%CI for the mean is shown for each subset and statistical significance is indicated (n.s. p≥0.05).

Figure s3

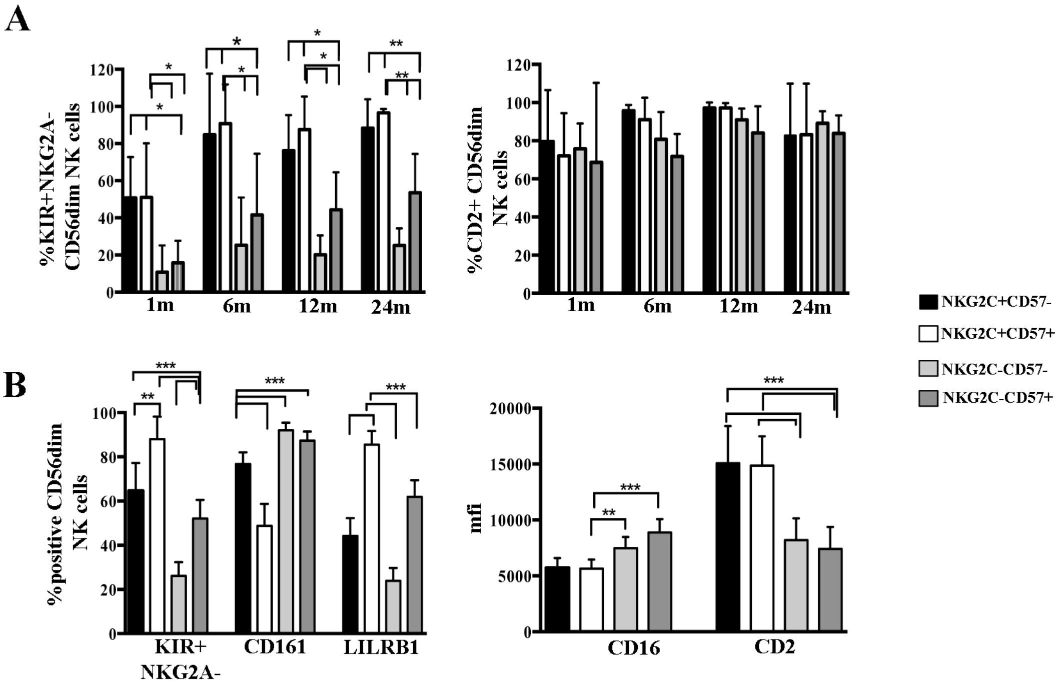

Figure s3. Surface phenotype of NKG2C/CD57 NK cells subsets in UCBT patients and HCMV^{+} healthy donors. PB-NK cells collected at different time points after UCBT from HCMV-reactivating patients (n=9) and from HCMV^{+} healthy donors (HD^{+}, n=36) were analyzed for the expression of the indicated surface markers, after gating on the different CD56^{dim} NK cell subsets identified by NKG2C and CD57. In (**A**) the percentage of KIR^{+}NKG2A^{-} (left panel) and of CD2^{+} (right panel) CD56^{dim} NK cells within the different NKG2C/CD57 subsets are shown in HCMV-infected UCBT patients from month 1 to 24. In **(B)** the percentage of KIR^{+}NKG2A^{-}, CD161^{+}, LILRB1^{+} CD56^{dim} NK cells (left panel), and the CD16 and CD2 median fluorescence intensity (right panel), are shown in HD^{+} in the different NKG2C/CD57 subsets. 95% CI for the mean percentage or median fluorescence intensity is reported and statistical significance is indicated (*p<0.05; ** p<0.01; *** p<0.001) in both (**A)** and (**B).**

Fig s4

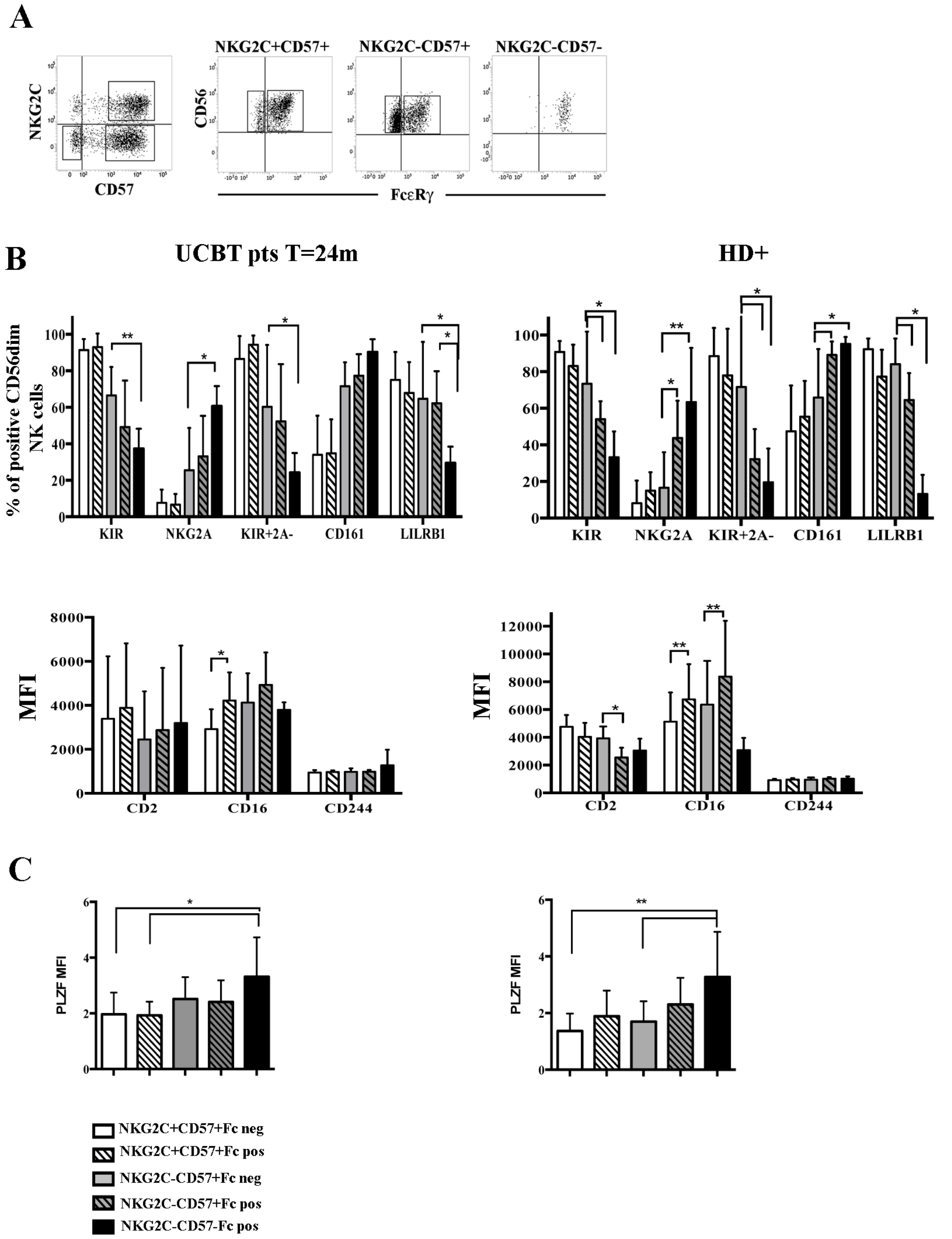

Figure s4. Surface expression of informative surface receptors in FcεRγ^{neg} and FcεRγ^{+} NK cells belonging to either NKG2C^{+}CD57^{+} or NKG2C^{-}CD57^{+} NK cell subset. (**A**) The gating strategy used to identify the different NK cells subsets is shown for a representative patient at 24 months after UCBT. After gating on CD56^{dim} NK cells, NKG2C^{+}CD57^{+} and NKG2C^{-}CD57^{+} NK cell subsets were identified and subsequently gated according to FcεRγ expression in NKG2C^{+}CD57^{+}FcεRγ^{+} or NKG2C^{+}CD57^{+}FcεRγ^{neg} and in NKG2C^{-}CD57^{+}FcεRγ^{+} or NKG2C^{-}CD57^{+}FcεRγ^{neg}, respectively. The NKG2C^{-}CD57^{-} NK cells, which are virtually all FcεRγ^{+}, were analyzed in parallel. In (**B**), upper panels, the indicated cell surface markers were evaluated in the different NK cells subsets described at the bottom of the figure, in HCMV-reactivating UCBT

patients at 24 months (left, n=8) and in selected HD$^+$ characterized by discrete amounts of the NKG2C$^-$CD57$^+$FcεRγ^{neg} (right, n=10). In the lower panels, CD2, CD16 and CD244 median fluorescence intensity (MFI) were analyzed in the same patients and HD$^+$. In (**C**) the median fluorescence intensity (MFI) of PLZF is shown in the different NK cells subsets for the same patients and HD$^+$ shown in (B). 95% CI for the median fluorescence intensity is shown and statistical significance is indicated (*p<0.05; ** p<0.01).

Figure s5

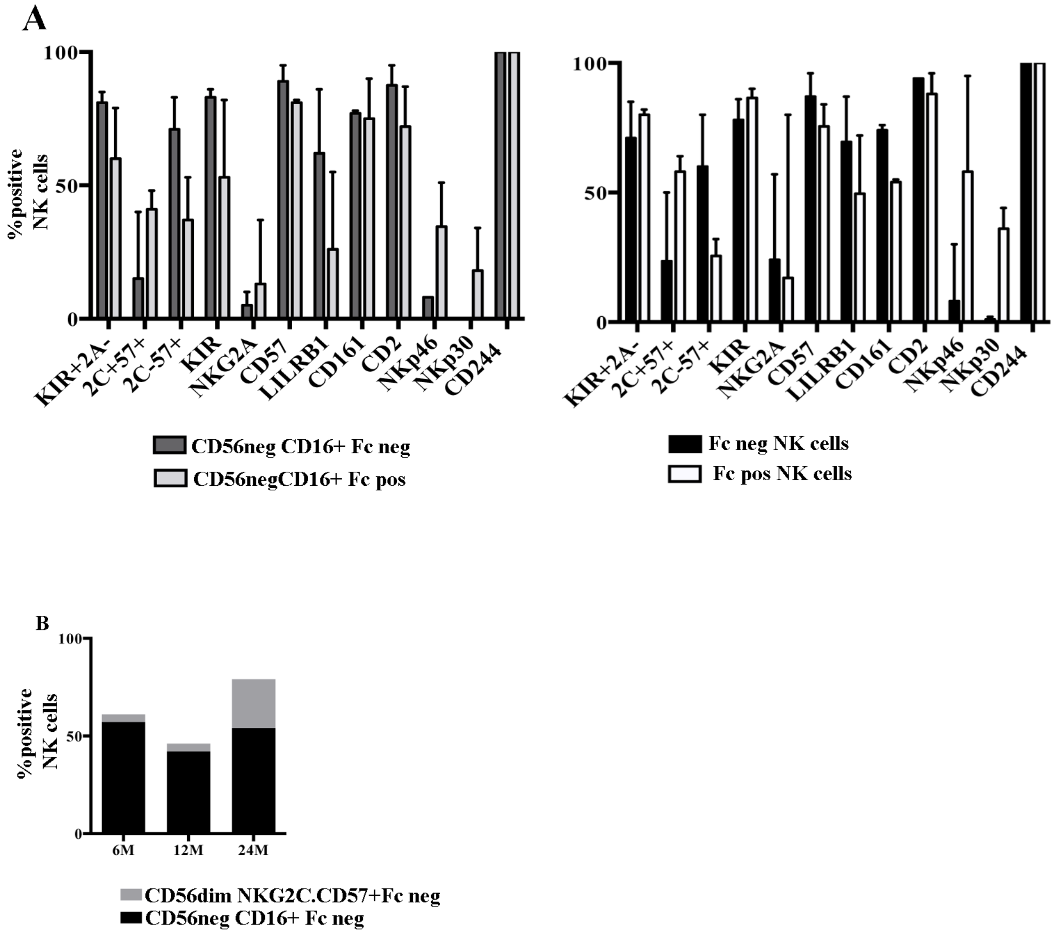

Figure s5. Comparison of the surface phenotype between FcεRγ^{neg} and FcεRγ$^+$ NK cells among lin- NK cells or within the CD56$^-$CD16^{bright} NK cell subset in HCMV-reactivating UCBT recipients

(A) The indicated cell surface markers or cell subsets were evaluated in FcεRγ^{neg} and FcεRγ$^+$ NK cell subsets in HCMV-reactivating UCBT patients at 24 months (n=4) as described in the figure

(left panel CD56^{-}CD16^{bright}, right panel lin- total NK cells). (**B**) The frequency of CD56^{-}CD16^{bright} FcεRγ^{neg} and of CD56^{dim}NKG2C^{-}CD57^{+}FcεRγ^{neg} within the whole FcεRγ^{neg} NK cell population was evaluated at 6, 12 and 24 months, in those HCMV-reactivating UCBT recipients developing significant amounts of the of CD56^{-}CD16^{bright} subset.

Figure s6

A

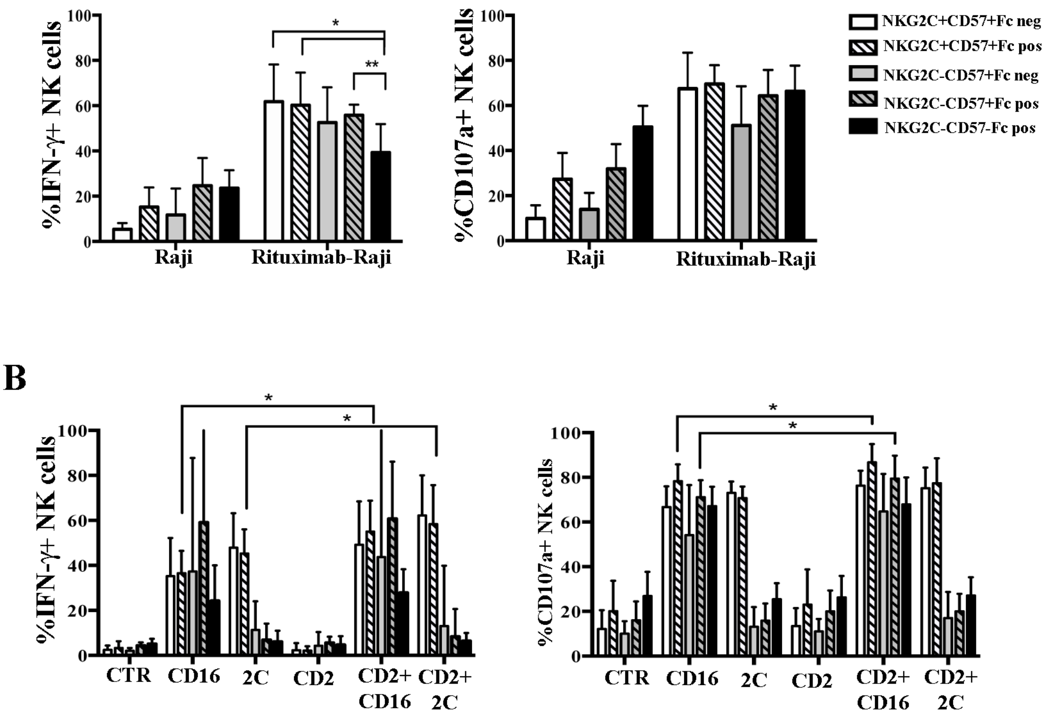

Figure s6. HCMV-induced FcεRγ^{neg} an FcεRγ^{+} NK cell subsets from HCMV^{+} healthy donors show comparable IFN-γ production and degranulation capabilities

The gating strategy used to identify the different NK cells subsets analyzed in functional assays is shown in fig. 6A (**A**) PBMC from HCMV+ healthy donors (n=10) were cultured overnight in the presence of rhIL-15. PBMC were then washed and cultured in the presence or in the absence of the Raji cell line, previously coated or not with the human anti-CD20 Rituximab (1 µg/ml), at an E:T ratio of 1:1, for 4 hours. Thereafter IFN-γ production (left panel) and CD107a mobilization (right panel) were evaluated for the indicated NK cells subsets as described in the figure. 95%CI for the mean of CD107a/IFN-γ positive NK cells is shown for each subset. Statistical significance is indicated (*p<0.05; ** p<0.01; *** p<0.001). In (**C**) after overnight culture, the same cells were incubated with the FcγR^{+} murine c815ell line p either in the presence or in the absence of anti-CD16, anti-NKG2C, anti-CD2 mAbs alone or in combination. CTR indicates cells cultured in the

presence of p815 and in the absence of mAbs. PBMC alone were virtually IFN-γ and CD107a negative in all subsets and were not depicted.

Figure s7

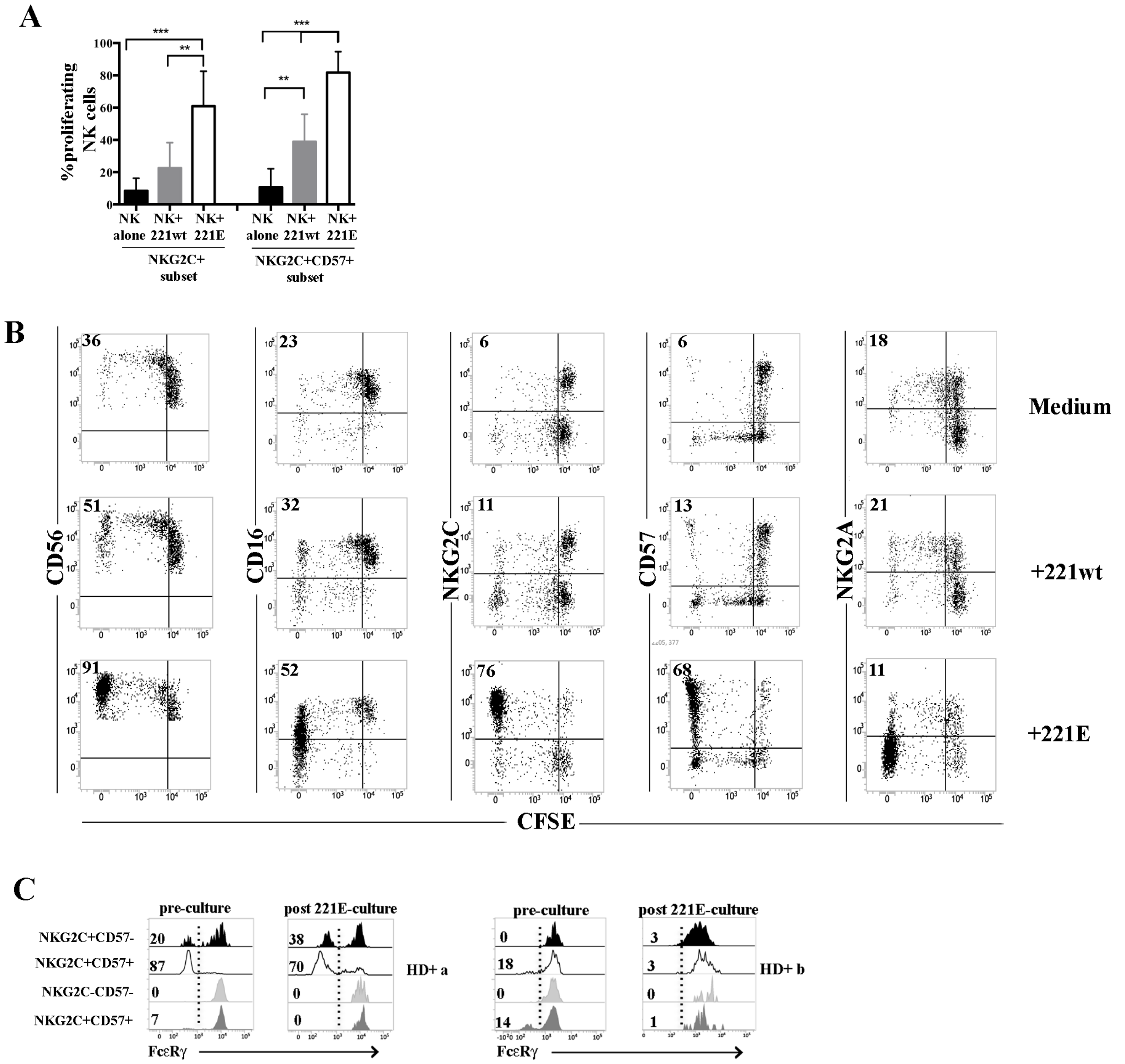

Figure s7. NKG2C$^+$ (CD57$^+$) from HCMV$^+$ healthy donors proliferate efficiently in response to NKG2C stimulation independently from FcεRγ expression. PBMC from HCMV$^+$ healthy donors (n=10) were CFSE-labeled and cultured in the presence or in the absence of 221wt or 221.AEH (indicated as 221E). FACS analyses on NK cells were performed as described at 7, 10, 14 days of co-culture, by gating on CD56$^+$ lin$^-$ lymphocytes. In (**A**) the percentage of proliferating (i.e. undergoing CFSE dilution) NKG2C$^+$ or NKG2C+CD57$^+$ NK cells in the different culture conditions are depicted (black bars: NK alone; grey bars: NK+221wt; white bars: NK+221E) at day 10 of coculture. 95%CI for the mean and statistical significance is indicated (** p<0.01; *** p<0.001). (**B**) The expression of the indicated markers is shown on NK cells from a representative

HCMV-infected UCBT patient in the different culture conditions at day 10. NK cell proliferation is determined by CFSE dilution. Percentages of proliferating NK cells are indicated in the upper left quadrant. In (**C**) FcεRγ expression is shown for the different NKG2C/CD57 NK cell subsets, identified as described above, in two representative HD^+ before and after 14 days of coculture in the presence of 221.AEH. Percentages of $FcεRγ^{neg}$ NK cells are indicated for each subset.
